# Human–AI collaboration for prehospital trauma triage: Designing the On Scene Injury Severity Prediction (OSISP) model as a clinical decision support system

**DOI:** 10.1177/20552076251403207

**Published:** 2025-12-12

**Authors:** Anna Bakidou, Magnus Andersson Hagiwara, Eunji Lee, Eva-Corina Caragounis, Bengt Arne Sjöqvist, Mattias Seth, Anders Jonsson, Stefan Candefjord

**Affiliations:** 1 Department of Electrical Engineering, 11248 Chalmers University of Technology, Gothenburg, Sweden; 2 PreHospen - Center for Prehospital Research, Faculty of Caring Science, Work Life and Social Welfare, 1802 University of Borås, Borås, Sweden; 3 Department of Surgery, Institute of Clinical Sciences, Sahlgrenska Academy, University of Gothenburg and Department of Surgery, Sahlgrenska University Hospital, Region Västra Götaland, Gothenburg, Sweden

**Keywords:** Artificial Intelligence (AI), Clinical Decision Support System (CDSS), customer journey map, digital health, eXplainable AI (XAI), field triage, human–AI collaboration, On Scene Injury Severity Prediction (OSISP), prehospital care, service design, trauma

## Abstract

**Objective:**

This study aims to advance the On Scene Injury Severity Prediction (OSISP), an Artificial Intelligence (AI)-based model, as a Clinical Decision Support System (CDSS) that supports Emergency Medical Service (EMS) personnel during on-scene assessment of adult trauma patients. The objectives are to explore the integration of OSISP with the prehospital trauma workflow and to refine the User Interface (UI) that communicates the predictions.

**Methods:**

Workflow integration was studied in a workshop by analysis of a customer journey map created by personnel with experience of working in the EMS setting (*n* = 8). Literature reviews were conducted to identify key factors enabling efficient human–AI collaboration and implementation options. Identified UI components derived from workshop and literature review findings were then evaluated and selected to refine the OSISP UI.

**Results:**

The workshop derived that OSISP is a service to be used on portable IT platforms as a second opinion, support for prioritization, and support during patient assessment. The literature reviews identified key content, characteristics, and goals of communicating predictions to users. The refined UI consisted of eight information components (prediction, entered predictors, missing predictors, and model details), and four functions (notification, exploration mode, and filtering of top three entered and missing predictors), to communicate the OSISP prediction.

**Conclusions:**

The refined OSISP UI has potential to integrate well into the clinical workflow during patient assessment, as well as enhance human–AI collaboration through customizable information when communicating predictions. However, usability testing of the OSISP UI is needed to ensure clinical utility.

## Introduction

In medical emergencies, such as trauma, minimizing time for providing optimal individual patient care is essential to increase the chance of survival and reduce the risk of disabilities.^1^ Trauma is the most common reason of death for young adults worldwide.^2^ For severely injured trauma patients, the nearest acute care hospital may not always be the adequate destination, and instead a more distant trauma center may be the preference for providing optimal care.^3^ It is therefore crucial for Emergency Medical Service (EMS) personnel to quickly decide on both initial care^1^ and appropriate transport destination.^1,3^

To make these critical decisions the EMS team must assess safety, scene situation, injury mechanisms, injury severity, care need, traffic situation and transportation time, as well as providing immediate care.^1^ All in all, the decision-making process is a difficult and time-pressuring task. The challenge is reflected in the literature, where a high proportion of patients are transported to care facilities with resources that do not match the actual care need.^4–6^ This may lead to a delay to optimal care, in particular for severely injured patients, increasing the risk of morbidity and mortality.^3^ Furthermore, excessive use of care resources for noncritical patients may impact care for critical patients.^3^

Artificial Intelligence (AI) has potential to help improve trauma care. In research studying epidemiological changes due to the COVID-19 pandemic, like reduced trauma volumes and specific injuries,^7^ AI has been used to explore impact of variables like age and place of residence on trauma types.^8^ Multiple studies demonstrate the ability to support management of traumatic hemorrhage by predicting mortality, need of transfusion, and injury severity.^9^ Furthermore, AI can predict the need for lifesaving interventions,^10^ and the risk of severely injured patients who can benefit from care at trauma centers.^11^ Integrating AI in Clinical Decision Support Systems (CDSS) therefore has potential to improve support given to EMS teams during assessment of trauma.

The prehospital challenges of assessing care needs and optimal transport destinations have previously been addressed by our research group, where digital technology is used to support the EMS personnel during the decision-making process. One example is the concept On Scene Injury Severity Prediction (OSISP). OSISP describes a CDSS to be used as a supporting tool by EMS personnel during the on-scene assessment of trauma patients. Based on mathematical models such as AI methods, OSISP uses patient information to predict the risk that the patient is severely injured. Initially, the concept was tested on retrospective motor vehicle crash data, indicating improved triage performance.^12–14^ Next, the OSISP scope was expanded to include all trauma incidents, using the Swedish Trauma Registry (SweTrau)^15^ as base, which shows promise for enhanced field triage.^16^

To advance OSISP toward clinical implementation, efforts are needed to develop a high-fidelity prototype and study usability in clinical practice. The Verified Innovation Process for Healthcare Solutions (VIPHS),^17^ a stepwise framework for the design, testing, utilization, and implementation of digital health tools and innovations, may be used as a guide. Building on previous work, documentation of the care process and design of the User Interface (UI) system remain to complete VIPHS step 1. These activities were initiated by Wallstén et al.,^18^ where a tablet-based UI prototype that incorporated eXplainable AI (XAI) features was proposed, including a prediction information page for communicating model predictions. When tested by six user participants, the prototype received high usability scores. For future research, the authors suggested focusing on enhancing specific aspects of communicating the OSISP predictions, for example, by providing explanations of feature importance to the user and when to notify the user.

The next step is to refine the care process and UI prototype by considering previous limitations, suggested future research, state of the art, and user feedback.^18^ The refinement of documenting the care process may be based on service design, a human-centered, collaborative, iterative, and sequential design approach to create holistic services that meets the need of reality.^19^ Customer journey mapping is a popular service design method used in healthcare,^20^ and visualizes how a customer experiences a service.^19^ By adopting customer journey mapping for the analysis of the care process, a deeper understanding of how to integrate the CDSS into the EMS personnel's workflow can be obtained.

Furthermore, XAI is important to consider for successful implementation of AI in healthcare.^21^ The refinement of the UI prototype will therefore focus on the prediction information page and be based on user feedback presented in reference^18^ and state of the art in human–AI communication. Examples of such communication items that have received attention are systematic model descriptions intended for clinical users,^22^ and incorporation of warning alerts when the model input does not match the model's intended use.^23^ There are several suggestions on how to communicate AI-based predictions to users, but it is also recognized that the design of an explainable interface needs to be customized for the task at hand.^24^ Instead of being used as general solutions, suggestions in the literature should therefore be evaluated on a case-by-case basis.

OSISP has not yet been designed as a CDSS to enable effective use in prehospital settings. This study bridges that gap by co-designing an interpretable, user-centred interface that supports EMS personnel in real-time decision making. The purpose of this paper is to further develop OSISP as a CDSS with emphasis on human–AI collaboration. The objectives are: (1) to identify critical information and decision points and explore the use of OSISP in the prehospital trauma workflow using customer journey mapping, and (2) to refine the prediction information page by suggesting an updated set of XAI features based on user feedback and current best practices. By completing VIPHS step 1, the ambition is to advance the OSISP concept and provide inspiration on how to address human–AI collaboration in emergency care.

## Methods

### Study design

This study was conducted in Sweden through a collaboration between Chalmers University of Technology, the University of Borås, the University of Gothenburg, Dedalus Sweden AB and an ambulance organization in the region of Västra Götaland. Information about the projects is given in the funding statement. The members had either research experience in digital health, technical experience in developing CDSS for the prehospital sector and in AI-based models, or clinical experience of working with trauma patients in Swedish prehospital settings or at trauma hospitals.

**Figure 1.**
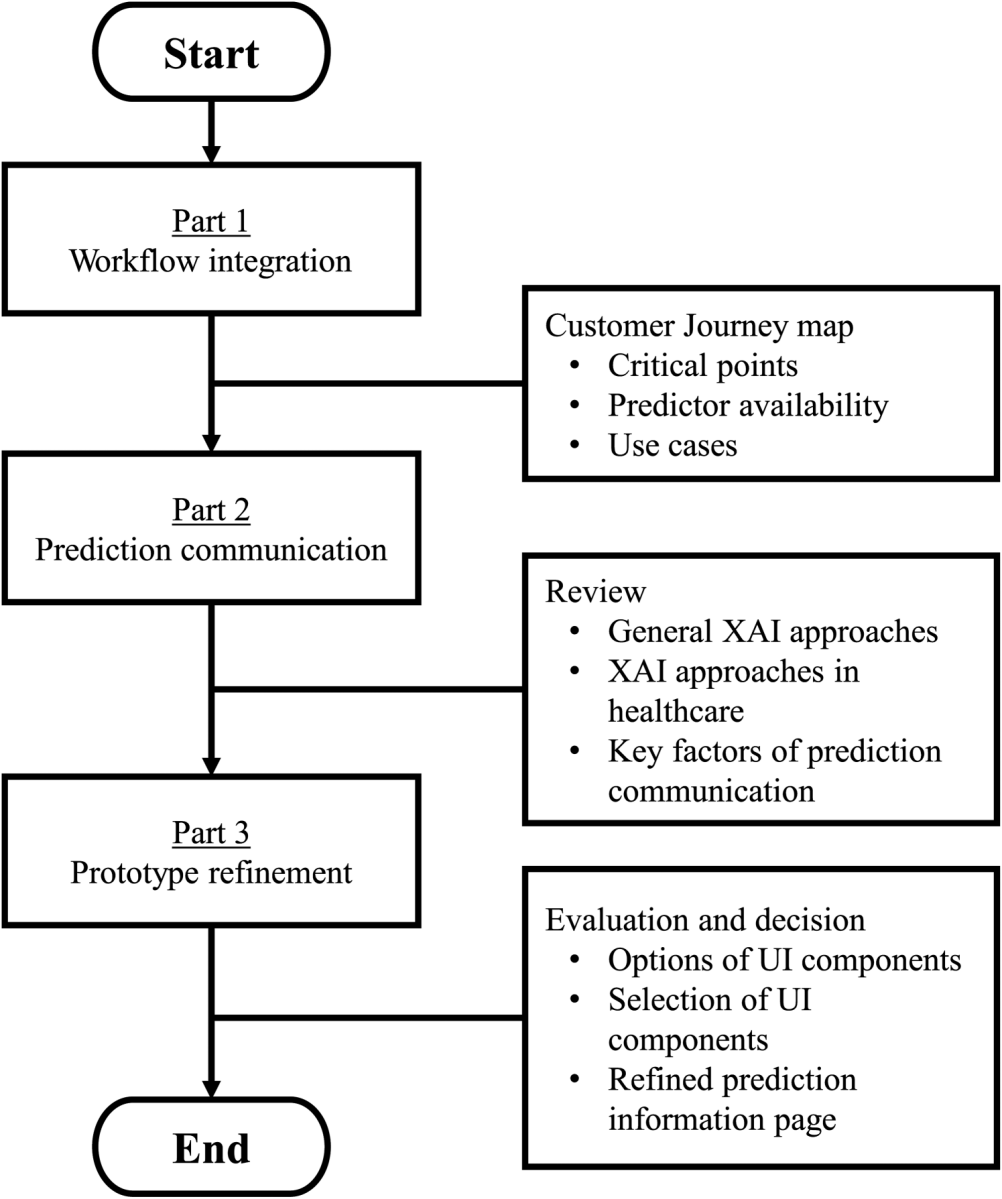
Overview of the method. The workflow integration was studied by creating and analyzing a customer journey map. State of the art for communicating predictions was studied by reviewing current XAI approaches and UI components. The findings were used to identify options of UI components to refine the OSISP prototype. OSISP: On Scene Injury Severity Prediction; UI: User Interface; XAI: eXplainable AI.

The study design was guided by the VIPHS framework.^17^ Because the development of OSISP is in an early design phase, the first step of VIPHS was targeted, which results in a blueprint describing the care process, technology proposal, system design proposal and theoretical testing and evaluations. Workflow integration and UI design proposal was the target for the presented study, complementing earlier studies.^16,18^ The method was divided into three parts (Figure 1). Part 1 explored the workflow integration. Part 2 reviewed XAI approaches in general and in healthcare. Part 3 refined the OSISP UI with an updated set of XAI features in the prediction information page, with suggestions on how to realize their functionality. This study focused on designing the OSISP as a CDSS, whereas empirical evaluation of the design proposal will be conducted in future work.

### Previous work and scope of study

The work in this study was based on previous results. Firstly, the technology proposal presented by Bakidou et al.^16^ was used to define the scope of the prototype. Secondly, the set of UI components, limitations, and suggestions for future research presented by Wallstén et al.^18^ were used as a foundation for refining the journey map and OSISP prototype. This study focused on identifying key information and features for effective human–AI collaboration. It did not address fundamental UI design elements, such as UI component shapes or placement.

### Part 1: Workflow integration

Customer journey mapping was used to analyze how to integrate OSISP with the users’ workflow. The map was co-created with intended end users in a full day (7 hours) workshop following the method outlined by Stickdorn et al.,^25^ with modifications (Table 1). The moderator (author AB) facilitated the workshop by providing instructions throughout the workshop. Critical information and decision points, and factors impacting decision making were concluded by participants during discussion of the customer journey map. The findings were used to identify appropriate workflow integration by an inductive thematic analysis of the transcribed discussions, conducted by a single researcher (author AB). To enrich the reporting of the workshop, quotations were translated to English and added.

**Table 1.** Details of the method used to study workflow integration, based on a modified method by Stickdorn et al.^25^.

Step	Description
1: Definition of the main actor and journey scope	*Main actor* EMS personnel working in ground ambulances *Scope* Patient scenario 1, overtriage: car running into a pedestrian at a pedestrian crossing Patient scenario 2, undertriage: elderly person falling at home
2: Preparation before the workshop	*Participant profile* EMS personnel with experience of working in Sweden Technicians with experience of developing prehospital technology *Invitation information* Full day workshop, participant information and informed consent
3: Initiation of workshop	*Activities (full workshop session)* Presentation of workshop and explanation of a customer journey example Division of participants into two groups and presentation of patient scenario 1
4: Identification of phases and steps	*Starting point of phases and steps (individual group session)* Six phases based on^26^: 1. Receiving the call, 2. Arriving at the scene, 3. On scene assessment and treatment, 4. Transport decision and departure, 5. En route assessment and treatment, 6. Handover Steps based on the process description created by author MAH, available in a supplemental file (Table S1 in Appendix A)
5: Iterations and refining of phases and steps	*Activity (individual group session)* Journey phases and steps reviewed by each group until agreement was reached
6: Adding journey perspectives to capture complexity	*Perspectives (individual group session)* Channels: Any means of communication (e.g. phone and radio)^25^ “What if”: “What could possibly go wrong?” (free text)^25^ Job to be done: What a product helps the customer to achieve (free text)^25^ Tools: Describes any tools that the user can use (e.g. equipment, protocols) Data availability: Data availability and how to collect it (e.g. vitals)
7: Merging maps and adding emotional perspective	*Activity (full workshop session)* Group presentations and feedback on generated group maps Discussion on similarities and differences and merging of maps Discussion on emotional aspects in each phase
8: Workshop discussion	*Extended discussion based on the customer journey map (full workshop session)* Changes in case of patient scenario 2 Additional factors: number of years working as EMS personnel, team collaboration, other organizational collaborations, and fear of consequences Locating critical information points (lacking or attention competing information) Locating critical decision points (when user must make an important decision) Presentation of the OSISP model Workflow integration: OSISP use cases and predictor availability
9: Follow up	*Activity (full workshop session)* Summary of workshop activities and results

EMS: Emergency Medical Service; OSISP: On Scene Injury Severity Prediction.

### Part 2: XAI approaches

To obtain an understanding of key factors that enable efficient human–AI collaboration, a literature review was conducted to find an overview of state of the art and recommendations on XAI in general and in healthcare in particular. The purpose of the review was to inform the design refinement of the OSISP UI. The methodology followed selected parts of full systematic/scoping reviews, as deemed appropriate for its purpose. The review was conducted by a single researcher (author AB) and the electronic databases used were PubMed, Scopus, ACM, and Web of Science. Snowballing was not conducted. The screening process was facilitated using the web application Rayyan (Rayyan Systems Inc).^27^

The review process was conducted as follows. First, relevant studies were identified by searching the selected databases. The search string “(artificial intelligence OR machine learning) AND (interfac* OR interact* OR explain* OR interpret* OR design* OR transpar*)” was applied on titles in the electronic databases. The search string used an asterisk (*) to capture variants of the words interface, interaction, explanation, interpretation, design, and transparency. Inclusion criteria were systematic and scoping review articles in English language. Because XAI is a rapidly evolving field, the search was limited to publications in 2024 to capture the most current XAI approaches. Second, the built-in function for detecting duplications in Rayyan was used to remove duplications. Third, abstracts were screened in Rayyan. Studies were included if the content described XAI approaches in general or applied in the healthcare sector. Papers related to drug development were excluded. Fourth, studies with full text access were included. Fifth, eligibility criteria were applied. Sixth, included studies were divided in two categories: general XAI approaches and XAI in healthcare. Lastly, for included studies, the following information items were charted in Microsoft Excel: XAI terminology, XAI taxonomy, and XAI techniques mentioned in more than one study. Additional aspects considered relevant for describing the state of the art were also documented, on which an inductive thematic analysis was conducted. The review did not consider data protection or integrity aspects. Based on the review findings, key contents and characteristics of prediction communication were identified.

### Part 3: Prototype refinement

A refined UI of the prediction information page that enhances human–AI collaboration was proposed based on the findings from parts 1 and 2 (Figure 1) and previous work on OSISP's UI.^18^ A complementary literature search was conducted to identify complementary implementation aspects for the OSISP UI. The search was conducted by a single researcher (author AB). The electronic databases were PubMed, Scopus, ACM, and Web of Science. E-journals that Chalmers University of Technology had access to through their digital database were also considered. Next, implementation options were evaluated, and a refined set of UI components were selected. Lastly, the updated set of UI components and identified key characteristics were used to refine the prediction information page in Microsoft PowerPoint.

### Ethical considerations

The study was conducted in accordance with the Declaration of Helsinki. Under the Swedish Ethical Review Act (SFS 2003:460), ethical approval was not required as no sensitive personal data or data about legal violations were processed. The data were managed in accordance with Chalmers University of Technology's policies and underwent a review by Chalmers Data Office. Written informed consent was obtained from all participants prior to participation in the workshop.

The workshop participants were employed by a project member organization and the time spent in workshops was part of their ordinary work time, and no additional incentive was used. Any sensitive personal data inadvertently collected from the workshop were excluded from the research material.

## Results

### Part 1: Workflow integration

This section presents findings from the workshop, where a customer journey map was created and analyzed.

#### Description of the workshop participants

The workshop was conducted by the moderator (author AB) and eight participants (involving authors MAH, ECC, and BAS). Participants had experience of working as EMS personnel (*n* = 3), EMS medical doctor (*n* = 2), trauma surgeon (*n* = 1), and industry representatives (*n* = 2). The majority of the participants were men (*n* = 6), the average age was 51 years, the clinical prehospital experience ranged from 8 to 17 years, and the experience of prehospital technology development ranged from 15 to 32 years. The participants had experience of working in various locations and the most common place was the Swedish region of Västra Götaland.

#### Customer journey map: Patient scenario 1

The participants assessed the journey maps of the workshop's two groups to be similar, with complementary aspects. The maps were therefore merged without modifications. The final customer journey map, accompanied by descriptions of each phase, is presented in a supplemental file (Table S2, Appendix B). Below follows extracted results connected to integrating OSISP into the workflow.

**Desired functionalities** Several functionalities were considered valuable to add to the workflow: a feature to see other units’ positions and statuses (however, this was found to be an available feature in some IT platforms); improved reporting to additional incoming units when arriving at site, with text format being preferred compared to audio; a structured approach for reflecting on what has been done; and a systematic feedback activity integrated within the workflow.

**Impact of patient scenario 2 on map** The second patient scenario (fall incident) was considered interesting since it represents a scenario where the EMS personnel are not sure whether the condition is critical or not. In these types of scenarios, it would be helpful with a CDSS.

“*… or maybe you need AI the most, those are the cases when you don't know if the patient is actually healthy or sick…* *will they get sick…* *so those who are very traumatized, it's quite easy, or you know what to do, but those who are on the borderline…”*

“*… and here is just… for example, is this a medical case or a traumatic case… you don't usually know that…”*

**Additional perspectives** The number of years in service was found to be important, and colleagues with a large amount of experience exhibit a calm that helps less experienced EMS personnel when encountering new situations, and experienced staff know how to care for patients when resources are restrained (e.g. if pharmaceuticals cannot be used). It was suggested that users may fear making wrong decisions and facing organizational punishments, and that they try to mitigate this risk by conducting additional assessments and treatments, even when it is not necessary. The perception of a friendly work environment improves performance, whereas an unfriendly work environment increases fear and reduces performance. Identified critical information and decision points are summarized in Table 2.

**Table 2.** Summary of critical points and availability of OSISP predictors throughout each phase of the prehospital trauma workflow.

Workflow phase	Critical point	First occurrence of OSISP predictors	Number of available OSISP predictors
1: Receiving the call	*Information* Expected situation	Age, Gender, Season of year, Intention of injury, Response time*, Mechanism of injury*	*N* = 6 (46%)
2: Arriving at the scene	*Decision* Quick or not quick? What to start with?	Response time*, Mechanism of injury*	*N* = 6 (46%)
3: On scene assessment and treatment	*Decision* What to do here and now? What to do during transportation? What to do at hospital?	Remaining predictors: Airway management, Location of injuries, Cardiac arrest, Dominating type of injuries, Glasgow Coma Scale motor response, Respiratory rate, Systolic blood pressure	*N* = 13 (100%)
4: Transport decision and departure	*Decision* Decision of transportation technique	*All predictors available*	*N* = 13 (100%)
5: En route assessment and treatment	*Information* Worsened condition *Decision* New decisions and destination. Prioritized measures	*All predictors available*	*N* = 13 (100%)
6: Handover	*Information* Information handover	*All predictors available*	*N* = 13 (100%)

Critical information points = lacking or attention competing information. Critical decision points = when the user must make an important decision. OSISP predictors are listed where they are likely to be collectable at first, and the number of available predictors in each phase.

*Predictors where first occurrence is uncertain.

OSISP: On Scene Injury Severity Prediction.

**Feedback on the OSISP concept** It was considered important that the EMS personnel make the decisions, not OSISP. As an example, the data entered to the model may be incorrect and result in incorrect predictions. Several use cases for OSISP were mentioned. First, it was considered useful as a second opinion, for example, when the recommendation differs from the opinion of the user, since the user may then decide to make a more thorough assessment. Secondly, if both the user and model agree, OSISP could be a support when prioritizing a patient, especially when a user considers reducing the priority to less urgent as this decision requires a solid basis for the decision. Excessive information is not appreciated as it may cause irritation. The idea of OSISP functioning in the background, with the ability to alert in case of a new or updated prediction as new data are entered, was appreciated. A similar functionality can be found in a current system for continuous monitoring, with the addition that alerts can be silenced by the EMS personnel if deemed unimportant.

“*I think, it's valuable when it thinks something different than what you yourself thought…* *then I think, okay, I've missed something here…* *because now I got a reservation here, I think this is trauma alert level one, and then I have to think about it a bit more…* *if it thinks the same as me, then it's just driving, then I don't focus on it any more…*”

“*… it would be a bit nice to say, yes but look, I think he's completely unharmed and this AI support also says he should be triaged as…*”

**OSISP predictor availability** Based on the journey map, the most likely first occurrences of the predictors were identified (Table 2). It was unclear if the predictors “Response time” and “Mechanism of injury” would be captured in phase 1 (receiving the call) or phase 2 (arriving at the scene), they are therefore listed in both phases. A complete set of predictors was found likely to be obtainable in phase 3 (on scene assessment and treatment), but a few of the predictors may be registered earlier, for example, gender and intention of injury.

**Summary of workflow integration** The medical purpose of OSISP is to predict if a patient is severely injured, and the intended user is EMS personnel working in ambulances. The OSISP is to be used on a portable IT platform that can be used both inside and outside of the ambulance. OSISP is currently only intended to be used in case of adult trauma, that is, patients older than 15 years. Predictions may be obtained from phase 1 (receiving the call), but a prediction based on a complete set of predictors is most likely available from phase 3 (on scene assessment and treatment) and onwards. OSISP may act as a second opinion, support prioritization of a patient, and support assessment in case of patients with conditions that are difficult to assess. Predictions are continuously updated as new data are entered, and the system alerts the user of any updates.

### Part 2: Prediction communication

**Figure 2.**
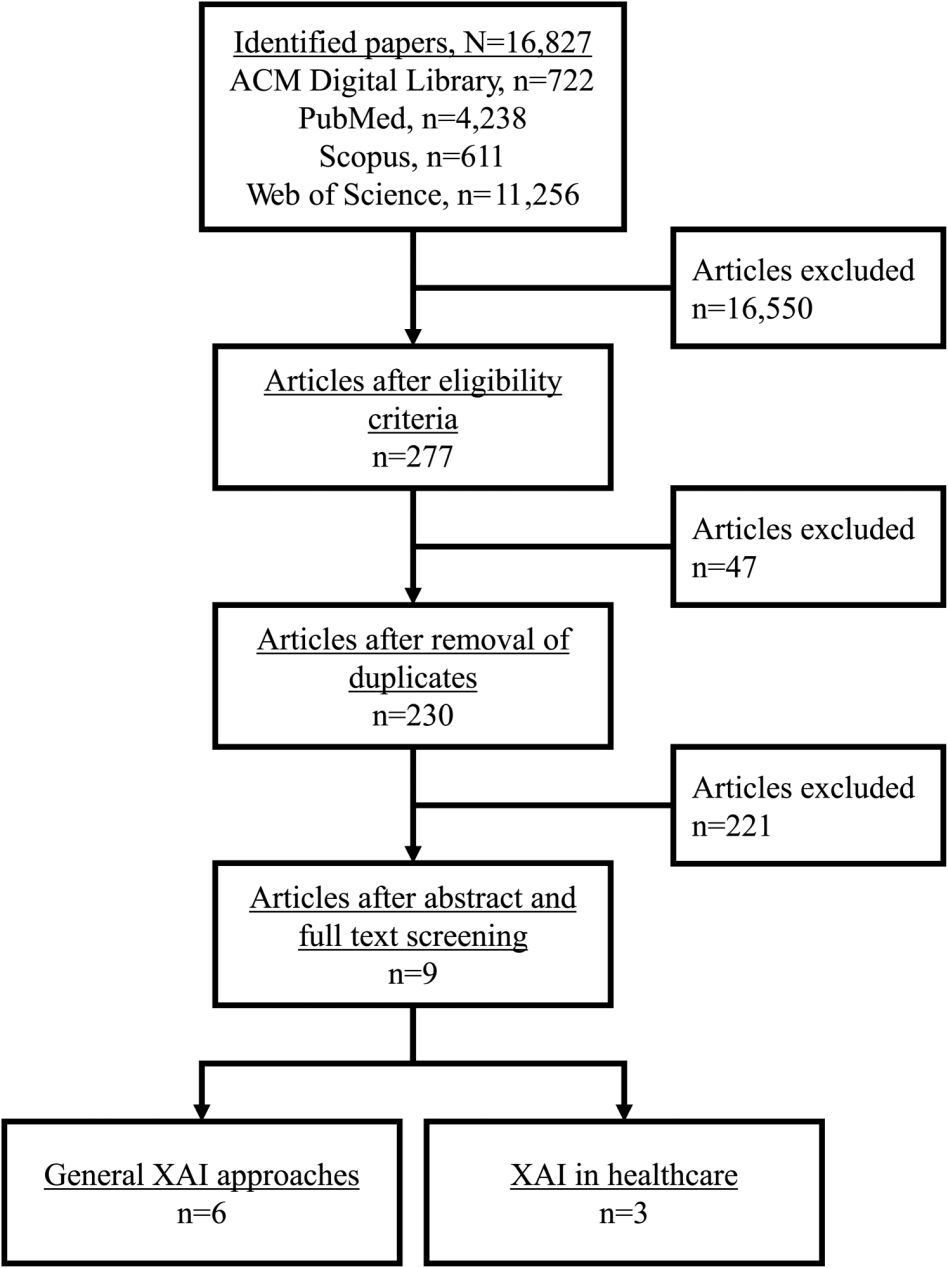
Review selection flowchart.

About 16,800 papers were identified in the initial search. After applying eligibility criteria, including filtering out nonreview papers and papers not published in 2024, nine unique reviews were included, where 6 reviews focused on XAI approaches in general^28–33^ and 3 reviews focused on XAI approaches applied in the healthcare sector.^34–36^ Figure 2 presents the selection flowchart, and charted information is presented in a supplemental file (Tables S3 and S4, Appendix C). XAI considerations identified as important for prediction communication are summarized in Table 3.

**Table 3.** Key findings on prediction communication based on literature review of eXplainable AI (XAI) approaches.

Consideration	Findings
Communication content	When prediction is uncertain Examples of interactions Why prediction is made (or not) When model succeeds or fails Detecting and reporting errors Hints about biases Contextual knowledge Permissible range Model details Development data details
Communication characteristics	Concise Contrastive Actionable Safe Interactive Scalable to other disciplines Adaptable to situation
Communication goals	Enable learning and reflection Enhance human capabilities Ensure human autonomy

#### Findings on general XAI approaches

**Algorithmic XAI** Much work have focused on developing algorithms to describe the functionality of models.^28,29,^^31–33^ The most frequently described techniques were Shapley Additive Explanations (SHAP) and Local Interpretable Model-Agnostic Explanations (LIME). A challenge is the trade-off between model performance and how well it can be understood.^28–33^ To manage this trade off, model-agnostic or hybrid approaches may be used.^32^

**Systematic descriptions** One approach is to systematically describe the data and model.^30^ Training data may be systematically reported using datasheets or data cards.^30^ A model can be described with model cards including for instance generalization expectations, accountability, or performance metrics and system errors.^30^

**Explanations** Another approach is to consider explanations.^28–31^^,33^ Explanations may be modeled as explorative processes, where users create understanding by reflecting on the topic.^30^ An example is to design interactive functionalities to trigger reflection instead of acceptance.^30^ Another approach is to provide explanation dialogs.^32^ Trust typically increases when explanations are provided,^31^ but explanations may cause negative consequences, for instance users acting against their self-interest, aligning their decisions with third parties, or being deceived by manipulated algorithms.^30^

**Customization** There is no one-size-fits-all solution,^30–33^ instead, a human-centered design adapted to the users’ expertise and needs is required.^29^ Customization is based on integration and content options, for instance why an explanation is needed,^30^ how to present it,^30^ when to present it,^30^ why a prediction was or wasn’t made,^28^ when the model succeeds or fails,^28^ when to trust the model,^28^ what the error is,^28^ what the bias is,^31,32^ what the risk of discrimination is,^31^ and how others have interacted with the system.^30^ Explanations should be concise,^28^ actionable,^30^ contextualized,^30,32^ contrastive,^30^ causal,^30^ and enable critique.^30^ Different goals may be targeted, such as enhancing human capabilities in decision making,^32^ ensuring human autonomy,^32^ and not overwhelming users with technical details.^30,31^

**Domain requirements** Critical decision systems, where an incorrect decision can lead to severe consequences, pose specific requirements.^33^ For such systems, safety is crucial and may be achieved by reducing bias, increasing robustness, and defining a model range.^33^ Further recommendations for applying AI in critical decision systems were to only use high performance models, quantify the model uncertainty, alert user when model is uncertain, and to conduct a manual check in case of differences in predictions and explanations between models.^33^

**Challenges** Several challenges were found. First, XAI research are often based on Western contexts with limited perspectives from the Global South.^30^ There is a lack of knowledge on how to build XAI systems,^28,30^ as well as how to make them scalable^28,32^ and adaptable.^32^ It is difficult to determine a sufficient level of explanation in different scenarios.^28,31^ More efforts are needed to make XAI systems that can detect and report errors^31^ and that are easy to use by nontechnical users.^28^

#### Findings on XAI approaches in healthcare

**Complementary aspects** The included papers were in line with the general findings, with a few complementary aspects. For instance, trust may be modeled as a quick and intuition-based human reasoning process.^35^ Furthermore, both the perception of the XAI's technical ability and emotions and beliefs tied to previous experiences may influence the perception of trust.^35^ Explanations were viewed as a tool that increases trust, but the impact depended on the user's knowledge in technical details of the model,^36^ as well as the structure and presentation of the explanation.^35^ Different clinical preferences on content and format of explanations were registered, such as case-specific logic of the model,^36^ visual form,^36^ and the combination of visual and textual explanations.^35^

**Challenges** XAI was considered important, but many questions on how to achieve successful implementation were unanswered. First, there are several challenges, for instance how to achieve an optimal level of trust,^35^ avoid blind trust,^35^ provide information that meet the end users’ needs,^35^ provide explanations for medical situations at the frontier of medicine,^35^ utilize clinicians’ expertise to verify the output,^36^ utilize XAI techniques that require background in mathematics and statistics,^36^ and how to visualize the XAI output.^36^ There is also a lack of knowledge in several areas, such as what information should be conveyed to aid decision making in healthcare, the structure of an effective explanation, and how to evaluate XAI methods.^36^

### Part 3: Prototype refinement

In this section, considerations and design options of the OSISP UI components are explored, evaluated and selected.

#### Complementary literature search

**Human factors** The decision-making process among paramedics has been found to be complex, and that the dual cognitive processing theory applies, i.e., that both intuitive (system 1) and problem-solving (system 2) processing are required.^37^ It was also found likely that novice paramedics more often use system 2 than 1 processing, while the opposite applies to experienced paramedics.^37^

**Prediction alert** A large body of research has studied what constitutes successful clinical alerts. Typically, designs should avoid alert fatigue, which causes users to override or ignore alerts.^38,39^ Examples on how to design successful alerts include easily interpreted interfaces,^39^ reduction of false alarms,^38,39^ appearance position at top of the screen,^39^ passive alerts in case of noncritical events,^38,39^ active alerts in case of critical events,^38,39^ frequency reduction by alert grouping,^38^ and vibrations or sound formats if visual notifications are not detected.^38^ An alert may also be triggered differently, for instance if a task has not been conducted within a specific time, or if a task has not been conducted until a certain step.^38^ Alert design should also consider how it fits within the alerting ecosystem, that is, how the newly added alert impacts the user when alerts from all the available tools are added.^40^ Specifically in the prehospital setting, users have requested to only be notified in case of critical events.^41^ Vibration and audio may be used as alert format, but there are reservations regarding these formats if patients and bystanders are close.^41^

**Prediction** In the case of a binary classifier, the outcome can be binary labeled or presented with the estimated probability of data point belonging to a class (also known as confidence score).^42^ For decision-making tasks, the prediction may be converted to a recommended action to increase usability.^42^ Clinicians also find it important to know what the prediction is based on, as well as reasons for and against the prediction.^43^ In triage tools currently used by EMS personnel, for instance the National Early Warning Score, Canadian Triage and Acuity Scale and American Emergency Severity Index,^44^ patient severity is categorized into discrete urgency levels. In one study on a prehospital model for predicting severe trauma, predictions were converted to actionable recommendations on where to transport the patient, with the result showing reduced undertriage.^45^

**Confidence level** It is important to communicate how certain the system is.^43^ Confidence scores may be calculated in several ways, for instance by using the likelihood, case-based reasoning,^46^ or the area under the receiver operating curve,^47^ however, the latter has not achieved a recommendable performance.^47^ Confidence may also be presented as uncertainty, with for instance predictive probabilities, predictive entropy, expected entropy, mutual information, or variation ratio.^48^ The format has an impact. A score number is not recommended as users may interpret it differently^43^ or not have the required knowledge to interpret scores correctly.^48^ Scores may be categorized,^43^ for instance as low and high confidence.^49^ Different transparency levels and colors can be used, but if visualized with the prediction, care must be taken to not confuse the outcome format with the confidence format.^42^ Also, the benefit of communicating the confidence may be reduced if other performance metrics are shown.^50^ Apart from calculation and formats, the relationship between the model's and the user's confidence impacts the resulting reliance on the system.^49,51^ For instance, confidence may be more difficult to grasp by nonexperts in case of high uncertainty,^52^ and increased task complexity may cause users to trust blindly on AI systems due to their uncertainty.^53^ Proper guidance on when the model is reliable and rationale for its prediction is therefore important.^52^ Lastly, confidence scores are only valuable if the model is calibrated.^48^ No studies on this topic were found from the prehospital setting.

**Model details** Model details may be systematically structured and documented with model cards that convey summaries of the general use.^22,54,55^ In critical decision systems it is crucial to know when the model can be used and not.^33^ A recent suggestion is to describe this information with a use card developed based on risk assessment according to the AI Act, where examples of content are intended purpose, type of product, application areas, primary actor, stakeholders and interests, success and condition, failure protection, and trigger.^56^ No studies on the topic were found from the prehospital setting.

**Data information** Data may be presented in different ways. One approach is as data cards with structured summaries.^57–60^ Another approach uses explorative data analysis information which is typically a subset of data card contents, for instance dimensionality, mean value, standard deviation, range, and missing samples.^61^ Data may also be described with question-based information, where each content item is connected to a question.^62^ No studies on the topic were found from the prehospital setting.

**Predictor contribution and ranking** Both SHAP and LIME are commonly used XAI techniques that can show local contributions, that is, new data points change each predictor's contribution. For SHAP, it is also common to average the contributions to global contributions, i.e., each predictor’s influence stays the same across new data points. SHAP has been reported to be more stable than LIME. No studies on the topic were found from the prehospital setting.

#### Evaluation and selection of UI components

The design should adapt to users utilizing both system 1 and 2 processing. To achieve this, the interface was designed with interactive cards that by default displays initial (system 1) information, with the option to access extended (system 2) information. Evaluation and selection of UI components are presented in Table 4, with indications of inclusion, modification and exclusion of previous OSISP UI components.^18^ A summary of all UI component and functions is presented in Table 5.

**Table 4.** Selection of new UI components.

UI component	Selected	Justification	Implementation
Notification^a^	Included	Selective function supporting familiarity, appropriate use and reflection	Active alert with yellow warning triangle in case of severely injured prediction, otherwise discrete alert
Risk prediction^a^	Modified	Supports familiarity and reduces cognitive load	C1: Discrete, color-coded prediction values ([unsure; not severely injured; severely injured])
Confidence level^a^	Modified	Reduces cognitive load and risk of inappropriate reliance	C1: Threshold to discrete prediction values, determined by healthcare organization
Explicit explanation^c^	Included	Concise summary reducing cognitive load	C2: Summary of most important content
Bias description^c^	Included	Supports safety and reflection	C2–C4: Information if predictor values were rare or common in training data
Predictors in favor/against condition^a^	Included	Contrastive and adaptive content supporting learning and reflection	C3–C4: Entered predictors, categorized if in favor or against condition based on SHAP values
Ranking of predictors^a^	Modified	Contrastive and adaptive content that supports reflection	C3–C4: Color-coded bars that show predictor contributions based on SHAP values
Filtered predictors^a^	Included	Selective and adaptive function supporting reflection	F2: Top three predictors for and against condition based on SHAP values
Missing predictors^a^	Included	Adaptive content supporting user action	C5: Predictors not yet entered
Ranking of missing predictors^a^	Modified	Adaptive content supporting user action	C5: Possible impact on prediction based on SHAP values, with priority to high-risk prediction
Filtered missing predictors	Included	Selective and adaptive function supporting user action	F3: Top three predictors, with priority to high-risk prediction
Model details^c^	Included	Supports safety and reduces cognitive load	C6: Model name C7: Model output C8: Permissible range
Exploration mode^c^	Included	Supports learning	F1: Enables modification of predictors without adding real data
Extended risk prediction information	Included	Supports learning and reflection	C1: Description of prediction, prediction alternatives, confidence ranges, outcome description and outcome thresholds
Extended summary information	Included	Supports learning and reflection	C2: Description of what content is included
Extended predictor information^b^	Included	Supports learning and reflection	C3–C5: Dataset name, dataset creator/owner, dataset language and location, data collection timeframe, number of instances, description of predictors, training and validation data distributions, missing information, if used as model input or output, support contact, and access to data card documentation
Extended model details	Included	Support learning and reflection	C6: Prediction and decision threshold, training and evaluation performance measures, who to contact in case of an issue, and access to model cards documentation
Extended model outcome information	Included	Supports learning and reflection	C7: Description of outcome, calculation of outcome and thresholds
Extended model use cases information	Included	Supports learning and reflection	C8: Description of permissible range and restrictions to consider
Risk prediction with predictor combination^b^	Excluded	Increases cognitive load	*Not implemented*
Model-specific techniques^b^	Excluded	Increases cognitive load	*Not implemented*

a Components from the first version of the OSISP UI presented in reference^18^.

b Future work suggested for the OSISP UI presented in reference^18^.

c New components suggestions.

CX/FX: component/function number; OSISP: On Scene Injury Severity Prediction; SHAP: Shapley Additive Explanations; UI: User Interface.

**Table 5.** Summary of UI components and functions.

UI component/function	Purpose	Design rationale
Component 1 (C1)	Displays the prediction: severely injured or not severely injured	Supports familiarity, learning and reflection. Reduces cognitive load and the risk of inappropriate reliance.
Component 2 (C2)	Provides a concise summary of the prediction, incorporating the most important details	Supports safety, learning and reflection. Reduces cognitive load.
Component 3 (C3)	Displays entered predictors for the patient being severely injured, ranked by their influence on the prediction	Supports safety, learning and reflection.
Component 4 (C4)	Displays entered predictors against the patient being severely injured, ranked by their influence on the prediction	Supports safety, learning and reflection.
Component 5 (C5)	Displays predictors not yet entered to the system, ranked by the influence for a severely injured prediction	Supports user action, learning and reflection.
Component 6 (C6)	Displays the name of the selected AI-model	Supports safety, learning and reflection. Reduces cognitive load.
Component 7 (C7)	Displays the outcomes that the selected AI model can produce	Supports safety, learning and reflection. Reduces cognitive load.
Component 8 (C8)	Displays the use cases for which the selected AI model is intended to be used for	Supports safety, learning and reflection. Reduces cognitive load.
Function 1 (F1)	Enables exploration mode, where users can test the impact of changing predictor values without changing the actual data	Supports learning.
Function 2 (F2)	Enables filtering of the top three entered predictors for and against condition	Supports reflection.
Function 3 (F3)	Enables filtering of the top three missing predictors	Supports user action.
Function 4 (F4)	Notifies the user that a prediction is available	Supports safety and user action. Reduces cognitive load.

AI: Artificial Intelligence; UI: User Interface.

#### Prediction information page refinement

**Figure 3.**
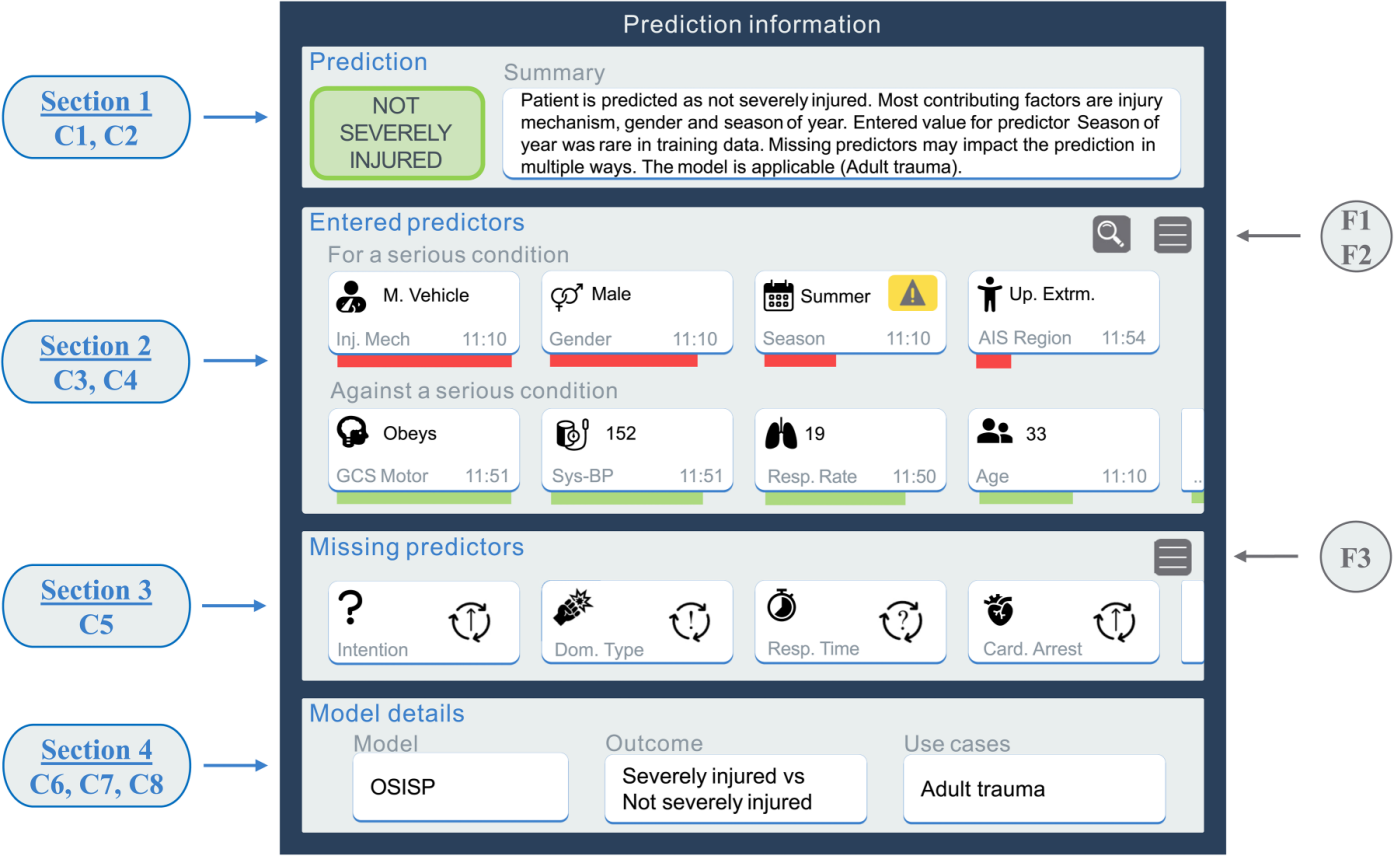
Refined OSISP prediction information page (initial information). The circles on the sides represent the UI component number: C1 = Risk prediction, C2 = Summary, C3 = Entered predictors for serious condition, C4 = Entered predictors against serious condition, C5 = Missing predictors, C6 = Model name, C7 = Model outcome, C8 = Use cases, F1 = Exploration mode, F2 = Filter for top 3 predictors for and against serious condition, F3 = Filter for top 3 missing predictors. OSISP: On Scene Injury Severity Prediction; UI: User Interface; M. Vehicle: Motor Vehicle; Inj. Mech: Injury Mechanism; Up. Extrm: Upper Extremity; AIS: Abbreviated Injury Scale; GCS: Glasgow Coma Scale; Sys-BP: Systolic Blood Pressure; Resp. Rate: Respiratory Rate; Dom. Type: Dominating Type of Injury; Resp. Time: Response Time; Card. Arrest: Cardiac Arrest.

The included UI components were used to refine the prediction information page of the OSISP UI into four sections: prediction, entered predictors, missing predictors, and model details. The prediction information page with initial information is shown in Figure 3 and additional design details are presented in a supplemental file (Appendix D).

## Discussion

This study has conducted customer journey mapping and literature reviews to explore considerations of integrating OSISP in the clinical workflow and communicating OSISP's prediction to end users, as well as proposed a refined UI. The results showed critical information and decision points, predictor collection points, use cases, common XAI approaches in general and in healthcare, and options of UI components. Reflection of the results is important to understand how OSISP may support the EMS personnel.

### Interpretation and implications of workflow integration

OSISP is intended to be used by any EMS personnel, from novices to experts, with varying education level and experience of working with AI or prehospital IT systems. Because novices may be defined as having less than a year of experience,^63^ the workshop participants represent experts, since the clinical experience was at least 8 years. Compared to experts, novices tend to use guidelines and system 2 thinking more^37^ and may therefore produce other use cases and design preferences. However, the critical information and decision points, and predictor collection points are considered independent of this factor as these were derived from standardized procedures. The workshop participants had mostly experience of working in a region where no digital platforms are yet available during prehospital patient assessment. In that regard, the participants are considered novices, which may cause their input to focus on usability rather than more detailed input on functionality and accuracy.^64^ Nevertheless, obtaining input primarily on usability was deemed appropriate during early phases of product design.

Running OSISP as a dynamic service, i.e., the prediction updates as new data are entered, was appreciated. This is in line with recent research on using AI-based CDSS in all steps leading to a diagnosis of sepsis.^65^ Practically, this would imply that OSISP can be used in all phases of the workflow, where early predictions (before reaching the patient) are based on alarm information. Notably, alarm information may be lacking or incorrect,^66^ causing two conflicting cases where OSISP may on one hand support mental preparation by complementing with an early prediction on the patient's condition, on the other hand add information that is based on less reliable data and in worst case be misleading. Another implication of early predictions is that a prediction will only be shown when the confidence is as high as safely required by the healthcare organization, therefore predictions may not be triggered early in the workflow due to too low confidence (e.g. due to fewer predictors available, Table 2). This may result in unavailable predictions from receiving the call until conducting the first on scene assessment. Moreover, the current OSISP model uses some predictors that cannot be unknown.^16^ Hence, if enabling early use of OSISP, the technology solution should be revised, and a warning of incorrect input data should be provided. As a result, when determining the confidence threshold for displaying predictions, healthcare organizations should consider the potential effects on both the model's performance (ratio of correct and incorrect predictions) and the availability of early predictions.

The current outcome of OSISP is a prediction whether the patient is severely injured or not. In a recent study, it has been proposed that the model outcome should be adaptable and recommend actions matching the steps leading to a diagnosis for successful human–AI collaboration.^65^ In view of this proposal, it could be argued that the current OSISP outcome does not adapt to the clinical decision-making steps of the workflow (Supplemental file, Appendix A). Also, the outcome is not explicitly stated in the assessment or treatment protocols used today, which may reduce the user's autonomy of following standardized procedures and thereby increase the user's dependence on the AI support. Consideration of alternative outcomes is therefore valuable and could be inspired by the critical decision points (Table 2) and use cases. Actionable outcomes supporting these activities could be obtained by mapping the prediction of severely injured or not to assessment and treatment policies, for instance recommending transport of predicted severely injured patients to trauma centers.^11^ However, recommendations on where to transport a patient often depends on carefully developed policies that consider the healthcare organization's resources. Because resources often differ between regions and nations, it is believed that an outcome on the patient's condition is more useful, as each healthcare organization can adapt it to their setting. Other more detailed alternatives of actionable outcomes could be recommended assessments, decisions and treatments, however, matching such outcomes would require extensive reviews by local clinical experts.

### Comparison of refined prediction information page with existing solutions

The refined prediction information page (Figure 3 and Supplemental file, Appendix D) is largely based on knowledge from scientific publications. Yet, the novelty of the field and rapid development of new methods may put stakeholders in industry as suitable representatives of state of the art. Since the literature review performed in the present study did not consider gray literature, such information could be overlooked. Dialogs with industry representatives indicate implementation challenges with best practices for regulatory compliance. A brief search of AI solutions at websites belonging to prominent companies delivering IT-solutions for EMS, for example, MobiMed by Ortivus AB (Stockholm, Sweden), amPHI by Dedalus (Milan, Italy), Paratus by OMDA (Oslo, Norway), EWA by Bliksund (Grimstad, Norway), and ZOLL emsCharts by ZOLL Data Systems (Broomfield, Colorado, US), did not result in any new insights about AI-based CDSS.

Most of the current field triage tools are rule based, i.e., the patient's priority is determined by checking if one or more criteria are fulfilled, typically predefined vital signs, injury type, and mechanisms of injury.^6^ There is an increasing body of literature presenting the potential benefit of using AI for predicting various trauma outcomes.^8–10^ However, to the authors’ knowledge, the literature on advancing these AI-models as CDSS is scarce and only one group from the Netherlands has been found to design their solution as a CDSS and advanced to prospective evaluations.^11,45^ That model classifies a patient as being severely injured or not, and was implemented as an application for smartphones and tablets named the trauma triage (TT) app.^45^ Predictors were managed page-by-page by either entering an integer (continuous predictors), selecting between yes and no (dichotomous predictors), or marking regions on a figure (injury predictors), with a resulting recommendation on transport destination.^45^

Comparison of current triage tools and the TT app with the refined OSISP UI presented here shows several differences. The OSISP UI is designed for tablets and does not provide an actionable outcome such as recommendation on transport destination. Information is not divided on separate pages, as entered input is displayed in the prediction information page, enabling the user to maintain a holistic picture of the situation without the need for information recall. The prediction information page was designed with intended end user and XAI in focus, resulting in extended information items (prediction, entered predictors, missing predictors, and model details) and functionalities for customization.

### Interpretation and implications of refined prediction information page

The scope of the prediction information page is interesting to reflect on. If considering the most basic format, AI-based CDSS could be formatted and viewed as advanced checklists to enable close comparison with current rule-based protocols. Yet, findings from the literature review suggest that additional information is needed to make AI predictions clinically useful (Table 3). A more advanced interface than a checklist is therefore deemed needed to enable efficient XAI communication. An example of such interface is the TT app.^45^ Although the work on the TT app has not addressed or evaluated XAI specifically, the interface presents a new approach for communicating predictors to users. When comparing the solution in the TT app with the proposed OSISP prediction information page, it is natural to question the scope of prediction communication. What is a reasonable scope? The question may be answered by reflecting on OSISP's position in the scientific debate on XAI and healthcare. It has been argued that black box models should not be used in a high stake domain like healthcare and that attempting to explain such models is misleading.^67^ Instead, inherently interpretable models are recommended since they produce faithful explanations and can achieve high predictive performance.^67^ By contrast, several of the reviewed XAI references point to the trade-off between interpretability and the predictive ability, list several examples of black box models with improved predictive performance, and emphasize the need of explanations when deploying AI in healthcare.

In light of this debate, the prediction information page may be considered a bridge. In the present study, the work was based on the proof-of-concept study, where models of varying complexity were tested and no significant difference in predictive ability was found.^16^ This could motivate the selection of the logistic regression model as it is inherently interpretable. However, besides conveying trust it is also important to not increase the cognitive load as end users already have multiple factors to consider during the decision making.^68^ With these aspects in mind, we believe the most important design principle is for the system to contain a uniform prediction information page applicable to any type of model, rather than focusing on whether the model is inherently interpretable or not. For instance, in the case of multiple CDSSs running in parallel, end users only need to learn how to use the prediction information page once to utilize any of the CDSSs. Employing a unified approach independent of model type is also in line with other XAI proposals, for instance systematic descriptions of models.^22,54,55^ To that end, the scope of the prediction information page is deemed appropriate.

A risk with the proposed OSISP UI may be increased cognitive load, as it consists of more information items and increased requirements for interaction compared to current field triage tools. Because novices tend to follow protocols strictly,^37^ the UI may cause novices to spend additional time navigating the UI compared to current checklists. This may increase the perceived task complexity, possibly causing automation bias, i.e., over-reliance on the system. In critical situations, an increased cognitive load may risk patient safety. At the same time, XAI is deemed needed for successful adoption in healthcare.^21^ A balance is therefore needed in our opinion, with a simple and intuitive design that minimizes the set of communication items and interactions, while providing understandable support that EMS personnel can use to make informed decisions. As no work studying XAI for prehospital use has been identified, finding a minimum set of UI components requires an iterative design process with intended end users involved.

Lastly, equal care should be provided to all. The refined OSISP UI has an increased possibility for customization. Users may then use OSISP with varying degree of efficiency, which raises the concern of its potential impact on the care provided. For instance, the extended information is a new proposal that intends to support novices, but may instead cause increased processing time if information is considered overwhelming. An alternative could be to remove customization functionalities so that only one navigation route results in the intended use. What is an appropriate level of design freedom remains to be answered as current findings on successful human–AI collaboration have not specifically targeted the prehospital setting. Verification and validation of OSISP and its UI with end users are therefore important to understand the potential impact of the proposed design on patient assessment and outcomes as well as clinical utility. This may be done using common design principles, for instance Nielsen's design principles.^69^

### Limitations

The customer journey map was generated based on the first patient scenario (traffic accident), with complementary discussions on potential changes in case of the second patient scenario (fall accident), making it possible that some aspects were missed when not constructing a separate customer journey map for the latter scenario. The workshop focused on ground ambulances, but OSISP may also be used in air and water ambulances. It is unclear how well the knowledge customer journey map may be transferred to these additional EMS services due to their different conditions. For instance, to the authors’ knowledge, air ambulances are usually staffed with a clinician with higher medical competence and have the option to transport patients over longer distances compared to ground ambulances. Water ambulances, on the other hand, are often staffed with people with lower levels of medical competence. Consequently, the generated customer journey map may lack perspectives to properly represent their workflow.

The literature review did not adapt the full methodology used for systematic or scoping reviews. This may impede the reproducibility of the findings. The review and search were also performed by a single author (author AB), which may cause biased results. However, the identified reviews largely overlapped, indicating that the most relevant content had been found. The terminology in XAI varies and may have caused some relevant literature to be missed. Only considering reviews during 2024 was decided to find current best practices, but may risk missing earlier XAI approaches. The explorative search to find inspiration for refining the UI may have missed important articles. None of the included literature on XAI targeted the prehospital setting specifically, therefore not all findings may be transferable.

The usability of the prediction information page was not tested in this study, which limits conclusions on how it will impact the EMS team's decision making during field triage. Furthermore, the flexible design allows EMS personnel to use the UI in a variety of ways, which may make conclusions on usability in future work challenging. The prediction information page was designed based on recommendations for successful human–AI collaboration based on findings from the workflow integration workshop and XAI review. Because the workshop was limited to ground ambulance teams and mainly included experienced participants from one region in Sweden, while the XAI literature did not address the prehospital setting, the prediction information page may need further refinement to enable efficient usage by all EMS teams. In addition, we acknowledge that technical barriers may impede implementation in current IT-platforms, but examining this in detail was beyond the scope of this study.

### Future work

The proposed workflow integration and prediction information page hold promise to support EMS personnel as a CDSS, but its potential must be validated in collaboration with EMS personnel to ensure that the functionalities support decision making during field triage as intended. Future work should therefore focus on conducting usability tests, where VIPHS^17^ should guide future activities. The findings from this study and previous OSISP activities resulted in a system design proposal, a first blueprint of step 1 of VIPHS. In the second step of VIPHS, the system design is tested in simulations with increased complexity. Thereby, future work should initiate the second step of VIPHS and conduct simplified simulations with EMS personnel, refine the system proposal if needed, and iteratively advance the complexity in the simulations until operational realism is reached. To ensure that all intended uses for OSISP have been identified and can be validated, it is important that future usability testing also involves participants with complementary experiences, i.e., EMS personnel with few years of experience and EMS personnel with experience of using IT platforms.

There are several functionalities that can be added to create a more holistic solution. First, the customer journey map should be expanded to cover perspectives of air and water ambulances. Next, EMS personnel may care for multiple patients at once. For such cases, ideally multiple CDSS for different conditions and outcomes could run simultaneously. An overarching model could be created to prioritize patients when resources are restrained based on predicted level of severity for individual patients, for example, for mass casualty incidents and military settings. Additional cognitive aspects may be interesting to study, for instance the perception of what is going on and higher system level impact from organizational and political-societal system levels. Adding these functionalities and aspects will make OSISP a more complete concept. For each refinement of OSISP, testing with end users, in controlled simulations with increasing complexity, is recommended to ensure usability.

To further advance OSISP, regulations, ethics and technical implementation are next to study. OSISP is considered to be an active medical device software since it is a service that analyses patient data and will be deployed on a digital platform. Special legal requirements need to be fulfilled for such device. To demonstrate that OSISP fulfills MDR, the process to obtain the CE-marking must be conducted, including the assessment of the risk class, product development, safety and performance evaluation, technical documentation, conformity assessment, and a plan for postmarket surveillance. Ensuring privacy and responsibility will be important ethical questions to address. As EMS personnel are responsible for their decisions when using current tools, a similar responsibility role is expected when OSISP is implemented. While the suppliers of OSISP must ensure good performance and patient safety according to regulations such as the AI Act in EU, the healthcare organizations implementing the CDSS must ensure that usage aligns with the supplier's documentation. The responsibility is therefore expected to be distributed across suppliers, EMS personnel, and healthcare organizations. Lastly, consulting suppliers of EMS IT systems on what UI components can be implemented is needed, for example, if the interactive functionalities are reasonable, and if standardized resources for exchanging healthcare information, such as Fast Health Interoperability Resources, exists to capture the extended information at the prediction information page.

## Conclusion

In this study, OSISP has been advanced as an AI-based CDSS by studying workflow integration, UI design and communication of predictions to end users. The customer journey map analysis found critical information and decision points throughout the prehospital workflow, which led to identification of several use cases for OSISP such as supporting assessment and prioritization of trauma patients, as well as being a second opinion. Workshop participants recommended OSISP to continuously update when new data are entered, and a complete set of predictors was found available from on scene assessment and treatment and forward in the workflow. Based on the literature reviews, key considerations for human–AI collaboration were identified, including recommended communication content, characteristics and goals, as well as implementation options. The findings were used to refine the UI page that communicates OSISP's predictions to end users, resulting in eight UI components and four UI functions with information on the prediction, entered predictors, missing predictors, and model details. The prediction information was divided into two layers: default information supporting experts, and extended information supporting novices or unsure users. Future work includes usability testing of the OSISP UI to ensure clinical utility. Further development of OSISP may be beneficial to support end users already when receiving the call. Lastly, this work has derived five design principles that may inspire development of other AI-based CDSS for healthcare in general, and prehospital settings in particular: (1) user-centered development (base design-phase on feedback from end users and workflow); (2) consistent UI (modular design usable by a variety of models); (3) contextual communication (holistic description with continuously updated information and adaptable notifications); (4) informed safety-nets against uncertainty (prediction explained and only communicated if above threshold and for intended use cases); and (5) flexible interaction (intuitive, customized, and actionable design).

## Supplemental Material

sj-docx-1-dhj-10.1177_20552076251403207 - Supplemental material for Human–AI collaboration for prehospital trauma triage: Designing the On Scene Injury Severity Prediction (OSISP) model as a clinical decision support system Supplemental material, sj-docx-1-dhj-10.1177_20552076251403207 for Human–AI collaboration for prehospital trauma triage: Designing the On Scene Injury Severity Prediction (OSISP) model as a clinical decision support system by Anna Bakidou, Magnus Andersson Hagiwara, Eunji Lee, Eva-Corina Caragounis, Bengt Arne Sjöqvist, Mattias Seth, Anders Jonsson and Stefan Candefjord in DIGITAL HEALTH

sj-pdf-2-dhj-10.1177_20552076251403207 - Supplemental material for Human–AI collaboration for prehospital trauma triage: Designing the On Scene Injury Severity Prediction (OSISP) model as a clinical decision support system Supplemental material, sj-pdf-2-dhj-10.1177_20552076251403207 for Human–AI collaboration for prehospital trauma triage: Designing the On Scene Injury Severity Prediction (OSISP) model as a clinical decision support system by Anna Bakidou, Magnus Andersson Hagiwara, Eunji Lee, Eva-Corina Caragounis, Bengt Arne Sjöqvist, Mattias Seth, Anders Jonsson and Stefan Candefjord in DIGITAL HEALTH

sj-pdf-3-dhj-10.1177_20552076251403207 - Supplemental material for Human–AI collaboration for prehospital trauma triage: Designing the On Scene Injury Severity Prediction (OSISP) model as a clinical decision support system Supplemental material, sj-pdf-3-dhj-10.1177_20552076251403207 for Human–AI collaboration for prehospital trauma triage: Designing the On Scene Injury Severity Prediction (OSISP) model as a clinical decision support system by Anna Bakidou, Magnus Andersson Hagiwara, Eunji Lee, Eva-Corina Caragounis, Bengt Arne Sjöqvist, Mattias Seth, Anders Jonsson and Stefan Candefjord in DIGITAL HEALTH

sj-pdf-4-dhj-10.1177_20552076251403207 - Supplemental material for Human–AI collaboration for prehospital trauma triage: Designing the On Scene Injury Severity Prediction (OSISP) model as a clinical decision support system Supplemental material, sj-pdf-4-dhj-10.1177_20552076251403207 for Human–AI collaboration for prehospital trauma triage: Designing the On Scene Injury Severity Prediction (OSISP) model as a clinical decision support system by Anna Bakidou, Magnus Andersson Hagiwara, Eunji Lee, Eva-Corina Caragounis, Bengt Arne Sjöqvist, Mattias Seth, Anders Jonsson and Stefan Candefjord in DIGITAL HEALTH

sj-pdf-5-dhj-10.1177_20552076251403207 - Supplemental material for Human–AI collaboration for prehospital trauma triage: Designing the On Scene Injury Severity Prediction (OSISP) model as a clinical decision support system Supplemental material, sj-pdf-5-dhj-10.1177_20552076251403207 for Human–AI collaboration for prehospital trauma triage: Designing the On Scene Injury Severity Prediction (OSISP) model as a clinical decision support system by Anna Bakidou, Magnus Andersson Hagiwara, Eunji Lee, Eva-Corina Caragounis, Bengt Arne Sjöqvist, Mattias Seth, Anders Jonsson and Stefan Candefjord in DIGITAL HEALTH
