# Supplementary material for: Human–AI collaboration for prehospital trauma triage: Designing the On Scene Injury Severity Prediction (OSISP) model as a clinical decision support system
Source: Digit Health. 2025 Dec 12;11:20552076251403207. doi: 10.1177/20552076251403207 (PMC12701220; doi:10.1177/20552076251403207)
Supplement: sj-docx-1-dhj-10.1177_20552076251403207 - Supplemental material for Human–AI collaboration for prehospital trauma triage: Designing the On Scene Injury Severity Prediction (OSISP) model as a clinical decision support system [file sj-docx-1-dhj-10.1177_20552076251403207.docx]

Title and description of supplemental material items

**Appendix A. Description of prehospital trauma workflow**

File format: .pdf

Title of data: Description of prehospital trauma workflow

Description of data: Process description of prehospital trauma workflow used to create the customer journey map.

**Appendix B. Customer Journey Map**

File format: .pdf

Title of data: Customer Journey Map

Description of data: Customer journey map created during the workshop and used to study workflow integration in part 1 of the method.

**Appendix C. Charted information of XAI approaches found from part 2 of the method**

File format: .pdf

Title of data: Findings from literature review on XAI approaches

Description of data: Charted information of XAI approaches found from the literature review on XAI approaches in part 2 of the method.

**Appendix D. Prediction information page proposal**

File format: .pdf

Title of data: Prediction information page proposal

Description of data: Visualization of prediction information page for communicating OSISP predictions, proposed in part 3 of the method.
