# Supplementary material for: Human–AI collaboration for prehospital trauma triage: Designing the On Scene Injury Severity Prediction (OSISP) model as a clinical decision support system
Source: Digit Health. 2025 Dec 12;11:20552076251403207. doi: 10.1177/20552076251403207 (PMC12701220; doi:10.1177/20552076251403207)
Supplement: sj-pdf-2-dhj-10.1177_20552076251403207 - Supplemental material for Human–AI collaboration for prehospital trauma triage: Designing the On Scene Injury Severity Prediction (OSISP) model as a clinical decision support system [file sj-pdf-2-dhj-10.1177_20552076251403207.pdf]

Appendix A. Description of prehospital trauma workflow

Process description of prehospital trauma workflow developed by author MAH. The description was used during creation of the customer journey map, where participants used the predefined phases and steps as a starting point when analysing the workflow.

Table S1. Process description of the prehospital trauma workflow. RAPS = a collaboration group including rescue services, ambulances, police and dispatch centrals (Swedish: Räddningstjänst, Ambulans, Polis, och SOS). SXABCDE = Safety Exsanguination Airway Breathing Circulation Disability Exposure.

Phase	Steps
1. Receiving the call	*Protective clothing<br>*Find the location<br>*Assess risks<br>*Collect more information, and plan<br>*Collaborate on RAPS channel and healthcare group channel
2. Arriving at the scene	*Assess risks<br>*Assess injury site and kinematics<br>*Report back<br>*Identify collaborating parties<br>*Establish healthcare management[a]<br>*Take report from medical officer[a]
3. On scene assessment and treatment	*Patient assessment according to SXABCDE<br>*Clinical decision-making (how urgent? Actions on site/actions en route? Unloading/transfer to ambulance)<br>*Interventions (bleeding control, airway, breathing, spinal motion restriction, pain relief?)
4. Transport decision and departure	*Transport decision (Level of care? Which hospital? Best route?)<br>*Reevaluation SXABCDE<br>*Decision on ambulance transport techniques
5. En route assessment and treatment	*Reevaluation SXABCDE<br>*Assessment and treatment not done on site<br>*Monitoring of vital parameters<br>*Reporting to receiving unit (phone). Type of trauma alert. Time of arrival. Request for on-site resources.
6. Handover	*Prepare and inform the patient about handover<br>*Structured report in the trauma room<br>*Patient record writing digitally<br>*Restore ambulance and equipment<br>*Reflection

[a] applicable in case of larger incidents.
