# Supplementary material for: Human–AI collaboration for prehospital trauma triage: Designing the On Scene Injury Severity Prediction (OSISP) model as a clinical decision support system
Source: Digit Health. 2025 Dec 12;11:20552076251403207. doi: 10.1177/20552076251403207 (PMC12701220; doi:10.1177/20552076251403207)
Supplement: sj-pdf-3-dhj-10.1177_20552076251403207 - Supplemental material for Human–AI collaboration for prehospital trauma triage: Designing the On Scene Injury Severity Prediction (OSISP) model as a clinical decision support system [file sj-pdf-3-dhj-10.1177_20552076251403207.pdf]

Appendix B. Customer Journey Map

Customer journey map created during the workshop (Table S2), followed by a summary of each phase. In the customer journey map, the following abbreviations and terms are used:

- Caller = the person reporting the incident to the EMS.
- CBRNE = Chemical, Biological, Radiological, Nuclear, and Explosives.
- METHANE = Major incident declared, Exact location, Type of incident, Hazards, Access, Number and type of casualties, Emergency services present and required. A structure used to communicate the first on site assessment upon arriving at the site.
- MIST = Mechanism of injury/illness Injuries Signs Treatment.
- Rakel = RadioKommunikation för Effektiv Ledning (Swedish). The national communication system for actors in order, security, health and defence in Sweden.
- RAPS = Räddningstjänst, Ambulans, Polis, och SOS (Swedish). Collaboration group including rescue services, ambulances, police and dispatch centrals.
- SBAR = Situation, Background, Assessment, Recommendation.
- SXABCDE = Safety Exsanguination Airway Breathing Circulation Disability Exposure.

Table S2. Customer journey map representing the prehospital workflow of EMS personnel in case of a trauma incident. The five left-most columns represent perspectives analysed in the workshop: Channels (some form of communication channel, e.g., telephone and radio), "What if" (what could go wrong, free text field), Job to be done (what should the user achieve/do, free text field), Tools (tools that the user can use, e.g., equipment and protocols), and Data availability (what data is available and how is it recorded, e.g., vital signs and dominant type of injury). The rows represent each phase of the journey. The right-most column presents the identified emotions that could be experienced in each phase. User refers to the main actor, in this case the EMS personnel. Product, company and protocol names are written with italic font.

	Channels	"What if"	Job to be done	Tools	Data availability	Emotions
Phase 1: Receiving the call	**Protective clothing** What to wear is based on alarm information provided by the emergency alarm central, through Rakel. Garments remain the same throughout the year, but lining may be used during winter. Protection may be used in case of CBRNE or electric vehicles. **Find location** GPS coordinates are provided with alarm information via the vehicle's computer. However, the map is often not updated on road works and users may instead use *Google maps*, *Apple* or similar solutions to locate the site. **Assess risks** Users consider if another unit is already on the scene, information provided by the emergency alarm operators and possible threats. **Gather more information** If accessible, the social security number can add information from the patient record. Caller and video support may also be used.	**Protective clothing** Dressing is completed before arriving at the scene, otherwise too late. **Find the location** Users consider traffic situation, and risk of incorrect or inappropriate road directions given by map system. The caller may be used to find location, but is sometimes unavailable to talk to the emergency alarm operators. Map apps may be used on private phones if vehicle's is deficient. **Assess risks** Users consider CBRNE, truck, chemicals, electric vehicles, number of patients, and traffic situations.	**Protective clothing** If there is a need, get dressed before arriving at the scene. **Find the location** Find location using GPS, local knowledge, experience, and contact with caller. **Assess risks** Assess threat and risk based on experience and information in the alarm.	**Protective clothing** Guidelines for protective clothing. **Find the location** GPS and setting in vehicle's computer to enable driving on bike lanes and similar. Radio and phone may also be used. **Assess risks** Emergency alarm central and the caller.	**Assess risks** Pictures from the scene of the accident may be accessible from the emergency alarm central. They can also provide position, risks and some patient background. Caller can also be used as an information source. **Collect more information, and plan** The emergency alarm central, the patient record system, e.g., *Melior* by *Oracle Cerner*, and the caller are sources of data.	*Anticipation *Sceptical *Lack of information *Initial concern *Anxiety

	Channels	“What if”	Job to be done	Tools	Data availability	Emotions
Phase 2: Arriving at the scene	**Assess risks**<br>Users may consult bystanders and staff for more information. The users experience a risk when multiple channels are being used on the radio. If users are the first unit on site, the users send a report. If another unit is first on site, the users receive a report. It is the user in the passenger seat that manages the communication.<br>**Assess injury site and kinematics**<br>Users may consult bystanders. In some cases, another unit may have been first on site and can be consulted for more information.<br>**Reporting back**<br>If needed, a back report is communicated to RAPS or the healthcare management, often structured according to METHANE.<br>**Other**<br>Oral communication with healthcare management and reporting.	**Assess risks**<br>The information provided with the alarm may not correspond to the actual situation and users need to be able to mentally adapt to a quick change of situation. In case of ongoing deadly violence, users await the police and care is delayed. If the situation becomes threatening, users may take a step back, await police, or activate an alarm received by the police and other ambulance units. Important information provided in channels may be missed if focused on a task.<br>**Reporting back**<br>Needed if more units are required. In some situations, the environment, e.g., tunnels or heavy traffic, leads to poor audibility.	**Assess risks**<br>Risks are assessed with visual inspection and by listening to witnesses or bystanders. Control if priority of patient needs to be raised or lowered and communicate it in the report. Users control patient records and determine what information is important to know for the given situation.<br>**Assess injury site and kinematics**<br>Recognize and assess the injury site. This is done by studying the condition of the car, how long the brake tracks are, and asking questions to the witnesses. One user becomes responsible for providing medical care. In some cases, both users need to work on the patient.	**Assess risks**<br>Location of the incident, experience, emergency services, police, and witnesses.<br>**Assess injury site and kinematics**<br>Vision, when inspecting the car, and hearing, when listening to witnesses<br>**Reporting back**<br>Performed using Rakel.<br>**Identify collaborating parties**<br>Rakel and visual inspection of site.	**Reporting back**<br>Data related to status may be available through patient record systems, e.g., timestamps from *Paratus* by *CSAM*.<br>**Other**<br>Data may be sent to and from the car in case of an incident through the E-call functionality, which connects to the emergency alarm central. This information contains the location of the car, type of car and the phone number.	*Relief<br>*Focusing<br>*Calm<br>*Or opposite (to relief)

	Channels	"What if"	Job to be done	Tools	Data availability	Emotions
Phase 3: Patient assessment and treatment	**Patient assessment**<br>Oral communication within the team. Depending on the environment, shouting may be necessary. Communication can also happen with Rakel.<br><br>**Clinical decision-making**<br>For the clinical decision, users may call higher medical competence locally available by using their phone.	**Patient assessment**<br>The patient condition may be worse than expected, and more staff or competence may be needed. There is a risk that the patient is inaccessible, e.g., ill-placed, unconscious or affected by substances. Depending on the environment, it may be hard to communicate. There is a risk with physiological dynamics, e.g., the patient deteriorates and needs intubation.<br>**Clinical decision-making**<br>Depending on the environment, it may be hard to communicate.	**Patient assessment**<br>Conduct patient assessment according to SXABCDE, involving e.g., control of bleeding and airways. In case of a trapped patient in a car, assessment is delayed. In case of multiple patients, users need to perform triaging. If possible, the patient is connected to monitoring equipment.<br>**Interventions**<br>If needed, perform repositioning, give pain relief.	**Patient assessment**<br>Clinical assessment according to SXABCDE guideline. A saturation measurement tool may also be used.<br>**Clinical decision-making**<br>The emergency bag is standard to use. If users need to walk to places further away from the ambulance, e.g., to an apartment, more equipment is brought, e.g., an airway bag. A chief medical doctor may be consulted when deciding on prioritization and measures. Equipment can be accessed from the ambulance, vehicle with medical doctor, and from the rescue services. Additional tools include the Prehospital Trauma Life Support protocol and treatment guidelines.<br>**Interventions**<br>The SXABCDE algorithm, treatment guidelines and locally developed app that calculates doses for pharmaceuticals and contains a map support.	**Patient assessment**<br>Better information about the patient makes it easier to make decisions about what equipment to bring for the assessment.<br><br>**Clinical decision-making**<br>Data generated from the SXABCDE assessment. Users also obtain information whether the patient is critical or not, type of injury and medical history.	*Focus<br>*Safe<br>*Relief<br>*Intensive<br>*Frustration<br>*Irritated

	Channels	"What if"	Job to be done	Tools	Data availability	Emotions
Phase 4: Transport decision and departure	**Transport decision**<br>Emergency alarm central and best route according to GPS or other map support systems. The notification is communicated to the hospital when sitting in the ambulance.<br>**Other**<br>If ambulance is outside of a larger city, users may call a chief physician staffed helicopter, trauma chief physician on call. These contacts, with the addition of the medical doctor at the receiving hospital, may also be consulted about the level of care and the situation.	**Transport decision**<br>Users consider environmental factors, traffic situation, weather, if helicopter can land, and if there are resources at the receiving hospital.	**Transport decision**<br>Assess transport destination and the patient record.<br>**Reevaluation**<br>Re-evaluate condition of patient.	**Transport decision**<br>App with information about trauma organization and care level structuring. Information may also be available in the patient record systems, e.g., *Paratus* by *CSAM*. Users may use their phone to call for higher medical competence.<br>**Reevaluation**<br>Treatment guidelines. While working with the patient, it is impractical to look at the phone or an app. Monitoring equipment may be used to monitor vitals, e.g., *Corpuls*.<br>**Decision on ambulance transport technicians**<br>Guidelines for spinal movement restriction.	**Reevaluation**<br>Vital parameters become available when redoing the patient assessment, e.g., through *Corpuls*.<br>**Decision on ambulance transport techniques**<br>Information on what technique to select is available in a guideline.	*Calm<br>*Relief<br>*Security<br>*Intensive<br>*Cheered up<br>*Nice<br>*Anticipation<br>*Frustration

	Channels	"What if"	Job to be done	Tools	Data availability	Emotions
Phase 5: En route assessment and treatment	**Reevaluation SXABCDE**<br>Dialogue within the team is important.<br>Closed loop and deepened interview of medical history.	**Reevaluation**<br>Re-evaluation is considered very important to accommodate for the risk of the patient deteriorating. Deficient communication within the team is a risk, and that users perceive the situation differently.<br>**Monitoring of vital parameters**<br>Users consider if the measurements taken with equipment are reliable or not, e.g., faulty equipment.<br>**Reporting to receiving unit**<br>Deficient communication with the receiving unit may take place.	**Reevaluation**<br>Re-evaluation according to guidelines.<br>**Assessment and treatment not done on site**<br>Assessment and treatment. During the transport, users also re-evaluate the transport destination.<br>**Monitoring of vital parameters**<br>If needed, the patient may be connected for continuous monitoring, but it leads to additional work and users need to enter the information in the patient record.<br>**Reporting to receiving unit**<br>Reporting is done by phone. There is a risk that all information collected from phase 1 (receiving the call) to phase 6 (handing over patient) is not included in the hand-over report to the receiving hospital. There is also a risk that the receiver has not understood the situation.	**Assessment and treatment not done on site**<br>Tool to measure pupil diameter, *Corpuls* for vital parameters, equipment for ultrasound (only in medical doctor equipped vehicles), tools to measure blood gas like pCO_2, and tool to measure lactate.<br>**Monitoring of vital parameters**<br>Monitoring equipment and *Corpuls*.	**Assessment and treatment not done on site**<br>Data from interviews on medical history. Data is also available on vital parameters from *Corpuls*.<br>**Monitoring of vital parameters**<br>Data from continuous monitoring.	*Calm<br>*Relief<br>*Security<br>*Intensive<br>*Cheered up<br>*Nice<br>*Anticipation<br>*Frustration<br>*Intensively<br>*Stress

	Channels	"What if"	Job to be done	Tools	Data availability	Emotions
Phase 6: Handover	**Prepare and inform the patient**<br>Inform the patient orally about the work in the trauma room.<br>**Structured report in the trauma room**<br>The receiving unit may be contacted by phone before arrival. When arriving, the hand-over report is orally presented, and the written ambulance patient record page is handed over.	**Prepare and inform the patient**<br>There is a risk that the patient is not receptive, for instance if being unconscious.<br>**Structured report in the trauma room**<br>There is a risk that the report that is handed over doesn't contain all the information. Also, there is a risk of the user becoming nervous or losing the thread while presenting the report to the trauma team, e.g., due to inexperience.	**Prepare and inform the patient**<br>Talk to patient before handover.<br>**Structured report in the trauma room**<br>Mentally prepare a structured report, which should include time stamps.<br>**Reflection**<br>Spontaneous activity after patient has been handed over. In case of doctor staffed vehicle, the doctors used to seek and communicate follow-up information on a patient if it was requested, however, this task is less conducted today.	**Prepare and inform the patient**<br>Voice for verbal communication.<br>**Structured report in the trauma room**<br>There are different report structures that could be used, for instance *SBAR* and *MIST*. There is also a possibility of obtaining a summary from *Corpuls*. Personal characteristics are also considered important tools to be able to deliver the report efficiently to the trauma team.	**Structured report in the trauma room**<br>Users want to include as much data as possible to reduce lost data. Data is available on the prehospital patient record page and *Corpuls*. It is also possible to obtain time stamps from Rakel.	*Nice<br>*Satisfaction<br>*Disappointment<br>*Anger<br>*Frustration<br>*Loss of control<br>*Performance anxiety<br>*Never quite satisfied

Summary of workflow phases

Phase 1. Receiving the call: The information provided with the alarm is crucial for mental preparation, decision on protective clothing, and need for additional support and collaboration actors. Physical maps used to be the standard approach to finding the location, but local knowledge combined with a digital map is the dominant approach today. EMS personnel focus on the most likely situation rather than preparing for all possible situations. Emotions that may be experienced were anticipation, scepticism, lack of information, initial concern, and anxiety.

Phase 2. Arriving at the scene: Healthcare management would not be needed for patient scenario 1. However, if needed, one of the EMS personnel in the unit first on site reviews the site and reports back to collaboration parties, e.g., police, rescue services or additional ambulance units. Information provided with the alarm may not always correspond to the actual situation, which makes it important that the first unit sends a back report with corrected alarm information to incoming units, so that they have a better opportunity for mental preparation. It was also emphasized that collaboration parties sometimes have more detailed and correct information compared to the ambulances, which causes confusion. Emotions that may be experienced were relief, focus, calmness, or opposing feelings to the ones mentioned.

Phase 3. On scene assessment and treatment: EMS personnel often bring an emergency and airway bag for the initial assessment, and need for additional equipment is based on walking distance between the ambulance and the patient and if additional units will arrive to support the team. In general, this phase was thought of as a focused session where a standardized assessment protocol will be used to identify if the patient is critically injured or not. If needed, EMS personnel have the possibility to call clinicians with higher medical education, for instance a medical doctor staffing the Regional Helicopter Service or a senior consultant in trauma surgery on-call at the Regional Trauma Center. If the patient is taken to the ambulance, more detailed assessments and interventions are enabled. Vital data may be generated, and if so, the participants preferred the data to be displayed as a graph rather than a list. Emotions that may be experienced were focus, security, relief, intensity, frustration and irritation.

Phase 4. Transport decision and departure: The most appropriate transport destination decision is a complex evaluation involving several factors with local and case specific restrictions, for instance if it is a traffic incident, if a helicopter can land, and what resources are available at the receiving hospital. To aid the decision, a clinician with a higher medical education may be consulted similarly as in phase 3. If the condition allows it, EMS personnel may conduct a deeper review of the medical history, perform additional assessments and re-evaluate the condition. There are many guidelines to aid the transportation technique decision of the patient to the ambulance, but the extensive information in the guidelines can make decision-making difficult. Emotions that may be experienced were calmness, relief, security, intensity, “cheered up”, nice, anticipation, and frustration.

Phase 5. En route assessment and treatment: Re-evaluation of the patient’s condition, being open-minded that the status may have changed, or that the transport destination may need to be changed are important considerations in this phase. Being in an ambulance enables to capture more data as more equipment is available. Communication is viewed as important, both within the team but also with the receiving medical facility. For the latter, there is a concern among EMS personnel of not being able to communicate the situation clearly enough, causing the receivers to not perceive the situation correctly. Emotions that may be experienced were calmness, relief, security, intensity, “cheered up”, nice, anticipation, frustration, and stress.

Phase 6. Handover: There was no discussion about the step of restoring the ambulance and equipment. Communicating to patient what will happen after the handover was considered important as patients are often unaware of the hospital procedures taking place at the emergency room, however, preparation may be hindered if the patient not responsive, for instance unconscious or in a challenging mental state. The handover report to the receiving trauma team was considered an important but challenging task that requires mental preparation and certain personal characteristics to be efficient. If the condition allows for it, a written patient record page will be handed over, however, the general structure of the page causes a variety of interpretations. For this reason, EMS personnel sometimes develop their own report for the handover. Reflection is not a defined task within the prehospital workflow and may spontaneously be initiated by the EMS personnel after the patient has been handed over. In some regions, teams involved in larger incidents are removed from further work to do a review on the incident. The handover should be completed within a certain time, but the time differs between regions. Emotions that may be experienced were nice, satisfaction, disappointment, anger, frustration, loss of control, performance anxiety, and never feeling satisfied.
