# Supplementary material for: Human–AI collaboration for prehospital trauma triage: Designing the On Scene Injury Severity Prediction (OSISP) model as a clinical decision support system
Source: Digit Health. 2025 Dec 12;11:20552076251403207. doi: 10.1177/20552076251403207 (PMC12701220; doi:10.1177/20552076251403207)
Supplement: sj-pdf-4-dhj-10.1177_20552076251403207 - Supplemental material for Human–AI collaboration for prehospital trauma triage: Designing the On Scene Injury Severity Prediction (OSISP) model as a clinical decision support system [file sj-pdf-4-dhj-10.1177_20552076251403207.pdf]

Appendix C. Findings from literature review on XAI approaches

Charted information of general XAI approaches

Table S3. Summary of general XAI terminology, taxonomy and techniques.

Category	Findings
XAI terminology	Explainable artificial intelligence, XAI<br>• "*…developing approaches to explain and make artificial systems understandable to human stakeholders…*"[30]<br>• "*…seeks to improve transparency and human comprehension of these systems, it is a machine learning method for addressing unclear AI systems decisions. It illustrates how an AI model functions and highlights its accuracy, fairness, and transparency…*"[28]<br>• "*…creation of human-comprehensible AI models that enable end-users to understand and trust their predictions while ensuring a high level of accuracy…*"[29]<br>• "*…an approach to develop open techniques that let consumers comprehend and trust the evolving AI systems while being able to govern them successfully…*"[32]<br>• "*…deals with making AI systems explainable, meaning to enable humans to understand the reasons behind a prediction…*"[33]<br>Explainability<br>• "*…the explanatory process for "opaque-box" or "black-box" models that are not inherently understandable…*" (also intelligibility)[30]<br>• "*…Understanding what a node means and how important it is to the performance of the model…*"[28]<br>• "*…characteristic of AI systems that gives humans understandable explanations for decisions made, outputs produced, and actions taken by the model in a transparent way…*"[32]<br>• "*…explainability involves deciphering the reasons (i.e., understanding why it operates)…*"[31]<br>Interpretability<br>• "*…describe the explanatory process for AI systems that are inherently understandable…* "(also traceability)[30]<br>• "*…ability of a model to infer cause and effect…*" and "*…capacity to reliably forecast a model's output without knowing the underlying causes…*"[28]<br>• "*… grasping the mechanics of a system without necessarily plunging into causality (i.e., comprehending how it operates)…*"[31]<br>Transparency<br>• "*… capacity to gain access to and examine the data used to train the model…*"[28]<br>• "*…inherently interpretable systems…" or "…promotes accountability…*"[30]<br>• "*…a model's ability to be understandable…*"[29]<br>• "*…To make the AI decision-making process clear and transparent…*"[32]<br>Accountability<br>• "*…ability to track and assign blame for an XAI system's decision…*"[28]<br>• "*… Making AI accountable for its deeds…*"[32]<br>Fairness<br>• "*…model is not favouring any one group or attribute over another…*"[28]<br>Human-centered explainable artificial intelligence, XAI

	• "*…techniques and principles that align with the explanatory needs of users…*" and "*…emphasis on a broader sociotechnical, personalized, and contextualized approach with a focus on the lay, non-expert user…*"[30]
XAI taxonomy	• Algorithmic XAI vs Human-Centered XAI[30]<br>• Model-agnostic vs model specific[31–33]<br>• Local vs global explanations[28,31,33]<br>• Ante hoc vs post hoc[32]<br>• Transparent vs post hoc interpretability[28]<br>• Intrinsic explainability vs post-hoc explanations[33]<br>• Data explainability, Model explainability, Post hoc explainability[30]<br>• Model-based, Representation-based, Post-hoc, Hybrid[31]<br>• Broad XAI, Explanatory AI (YAI), Evaluative AI[30]<br>• Glass/white box vs black boxes[28]<br>• Rule-based reasoning, Post hoc explanations, and Model-specific justifications[28]<br>• Rule-based explanations, Feature relevance methods, Visualization techniques, Natural language explanations[28]<br>• Visual Explainability, Rule-based Explainability, Case-based Explainability, Natural Language Explainability, Knowledge-based Explainability[29]<br>• Numeric Explanations, Rule-based Explanations, Visual Explanations, Textual Explanations, Mixed Explanations[32]<br>• Feature summary statistics, Visualization of feature summary, Model internals, Data points, Interpretable model[33]
Utilized techniques	Counterfactual explanations,[31,33] Decision trees,[31–33] Feature importance,[32,33] Fuzzy rules/logic,[32,33] "If-then" statements,[29,31,32] Integrated gradients,[28,33] Local Interpretable Model-Agnostic Explanations (LIME),[28,29,31–33] Linear regression,[31,33] Shapley Additive Explanations (SHAP),[28,29,31–33] Tree-based systems[29,32]

Charted information of XAI approaches in healthcare

Table S4. Summary of Healthcare XAI terminology, taxonomy and techniques.

Category	Findings
XAI terminology	XAI<br>• "*…methods which can be applied to black box models to generate interpretable explanations for their predictions…*"[34]<br>• "*…methods and techniques that craft AI applications comprehensible not just to domain experts or data scientists, but also to laypersons unfamiliar with AI intricacies…*"[36]<br>Explainability<br>• *"…crafting an interface that makes the inner workings of AI decision-making accessible and comprehensible to humans…"*[36]<br>Interpretability<br>• *"…providing human-understandable rules that govern a system's decision-making process…"*[36]<br>Transparency<br>• *"…designed to retain the high-performing predictive capabilities of ML/DL but have the interpretability of simpler models…"*[34]<br>• *"…models whose rationale can be interpreted or elucidated with ease…"*[36]<br>Trust<br>• *"…a decision-making shortcut, enabling the decision maker to select information while ignoring other information to simplify a complex decision …"*[35]
XAI taxonomy	• Intrinsic vs post hoc[34]

	• Model-specific vs model-agnostic[34,36]<br>• Individual, global, and feature/outcome relationships[34]<br>• Local, global, counterfactual, confidence, example-based[35]<br>• Feature-oriented, global, concept, surrogate, human-centric[36]<br>• Opaque/Black, grey, white[36]
Techniques utilized in healthcare	Counterfactual,[34,36] Feature importance,[34,36] Local Interpretable Model-Agnostic Explanations (LIME),[34,36] Partial dependency plots,[34,36] Rule-based,[34,36] Shapley Additive Explanations (SHAP)[34,36]
