# Supplementary material for: Human–AI collaboration for prehospital trauma triage: Designing the On Scene Injury Severity Prediction (OSISP) model as a clinical decision support system
Source: Digit Health. 2025 Dec 12;11:20552076251403207. doi: 10.1177/20552076251403207 (PMC12701220; doi:10.1177/20552076251403207)

Appendix D. Prediction information page proposal

This appendix presents the refined OSISP UI page, called prediction information page, that communicates predictions to end users. Each section below displays the different parts of the prediction information page with examples of data.

Prediction information page components and functions: Default information

Default view of the prediction information page. It consists of eight components (C1–C8) and three functions (F1–F3), organised into four sections: Prediction, Entered predictors, Missing predictors and Model details. Each component can be pressed to access extended information.

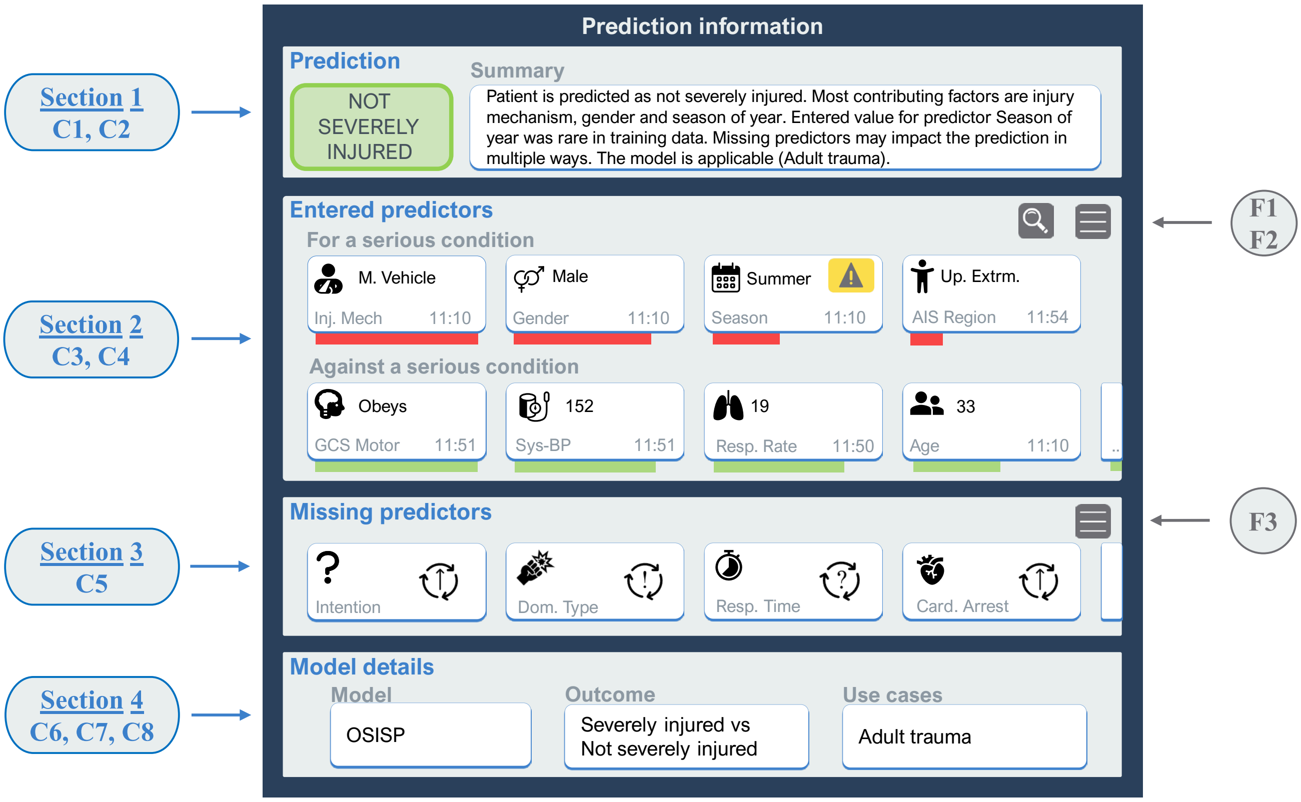

Component 1, Risk prediction (C1): Extended information

Component 1 displays the risk prediction, and if pressed, a pop-up box with extended information is accessed. The pop-up box is scrollable and may access information displayed in the numbered box 1.

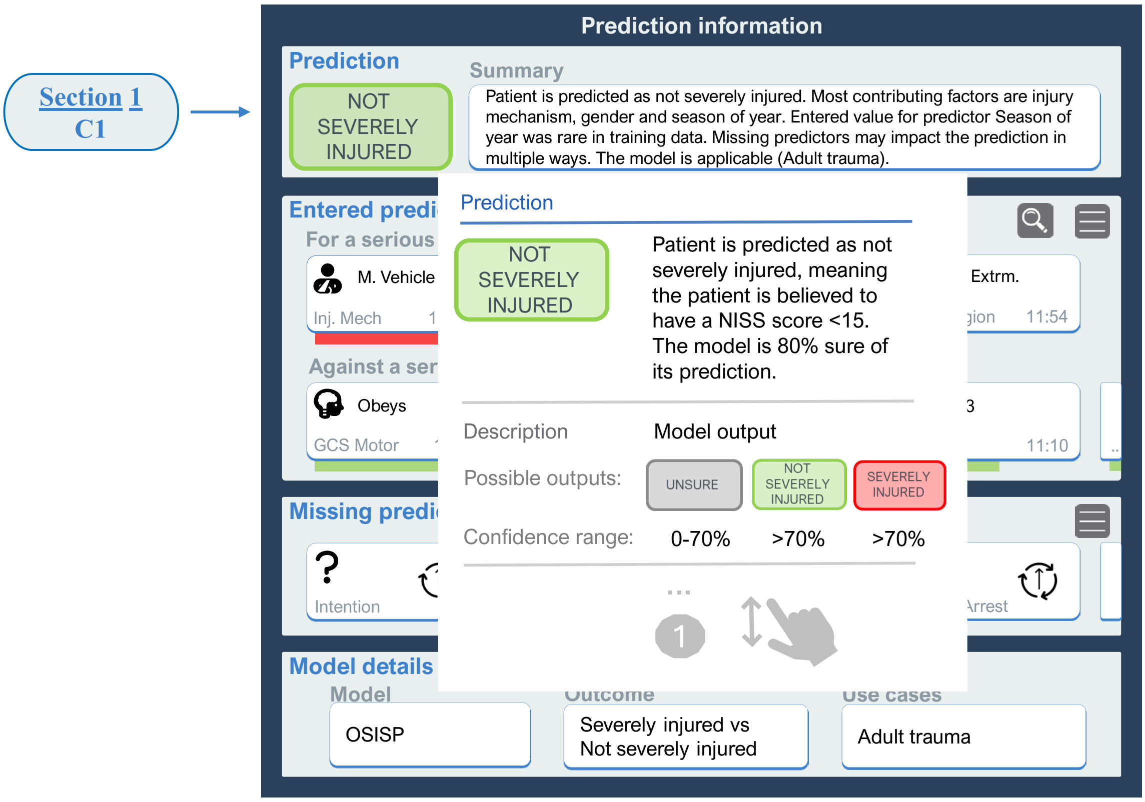

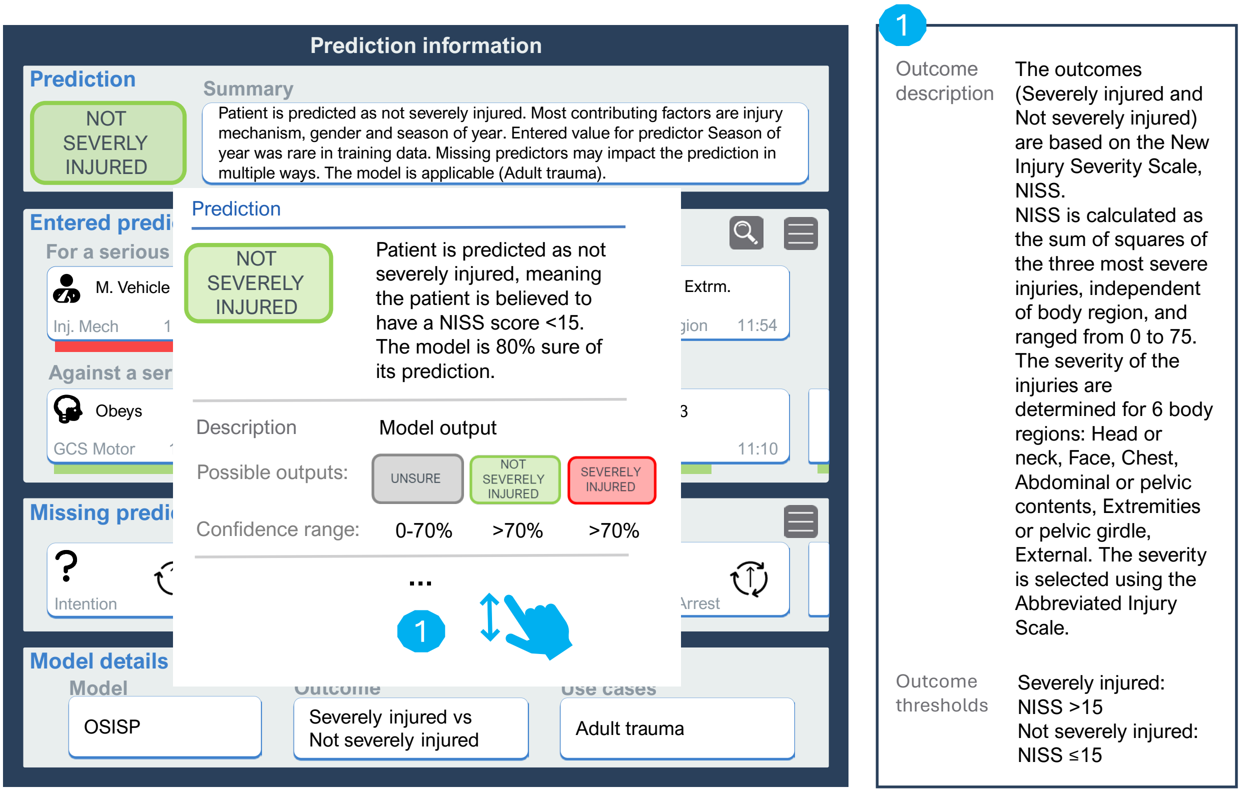

Component 2, Summary (C2): Extended information

Component 2 displays a text summary of the page, and if pressed, a pop-up box with extended information is accessed.

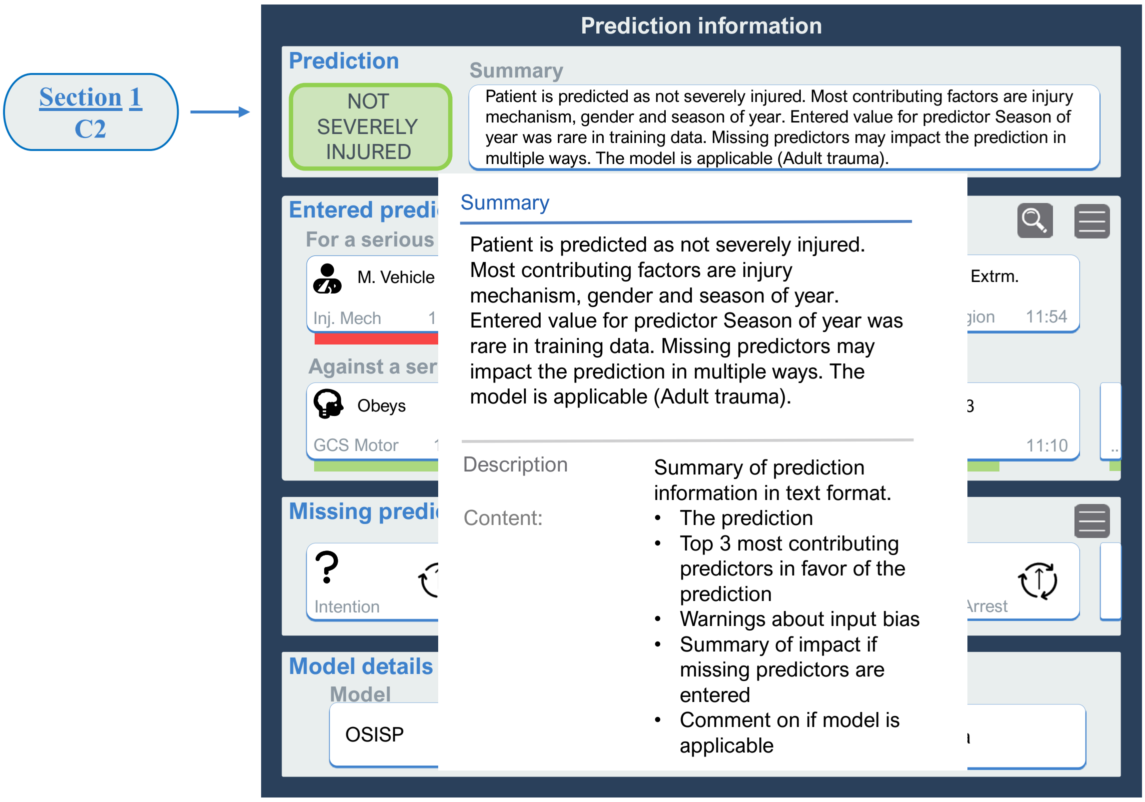

Component 3 and 4, Entered predictors (C3 and C4): Extended information

Components 3 and 4 display entered (first row) and missing (second row) predictors respectively, and if any predictor is pressed, a pop-up box with extended information is accessed. The initial pop-up box displays available and selected predictor values. If the information box is pressed, additional scrollable information is accessed, displayed in the numbered boxes 1–2. If an entered predictor value was rare in the training data, a warning will be communicated to the user.

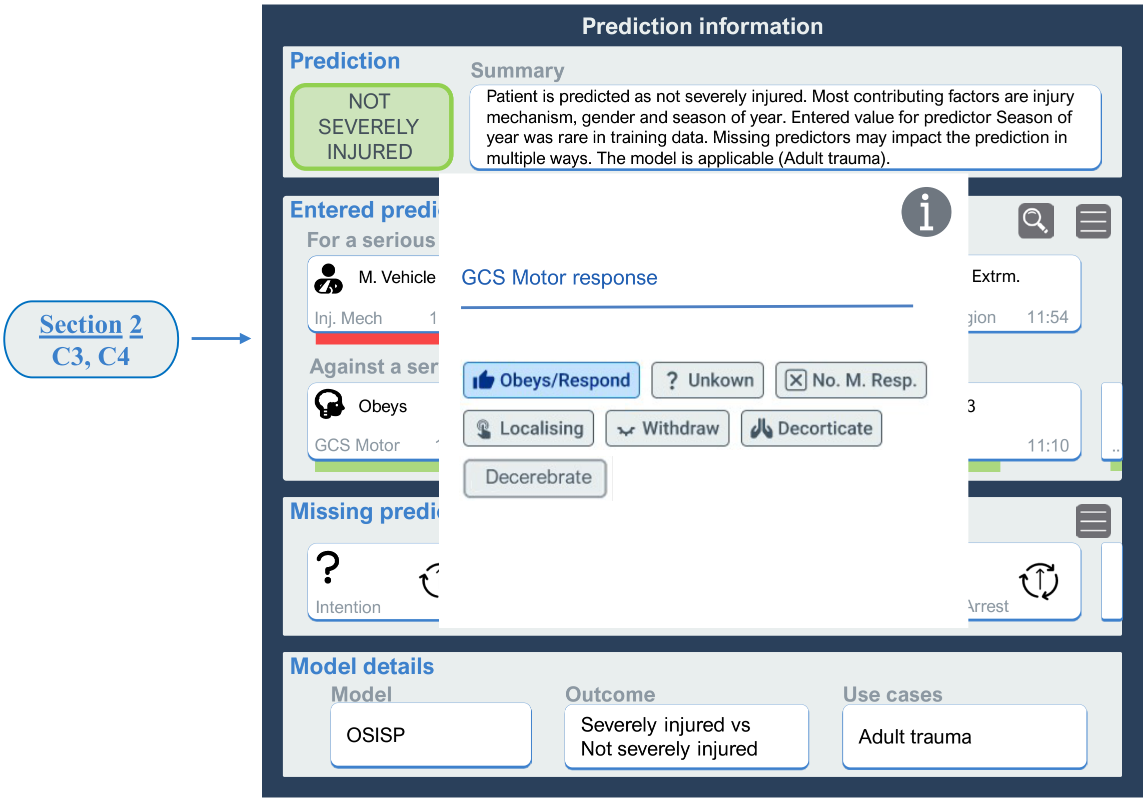
Section 2
C3, C4
Prediction information
Prediction
NOT SEVERELY INJURED
Summary
Patient is predicted as not severely injured. Most contributing factors are injury mechanism, gender and season of year. Entered value for predictor Season of year was rare in training data. Missing predictors may impact the prediction in multiple ways. The model is applicable (Adult trauma).
Entered predi
For a serious
M. Vehicle
Inj. Mech
Against a ser
Obeys
GCS Motor
Missing predi
Intention
GCS Motor response
Obeys/Respond
Unkown
No. M. Resp.
Localising
Withdraw
Decorticate
Decerebrate
Extrm.
11:54
11:10
Arrest
Model details
Model
OSISP
Outcome
Severely injured vs Not severely injured
Use cases
Adult trauma

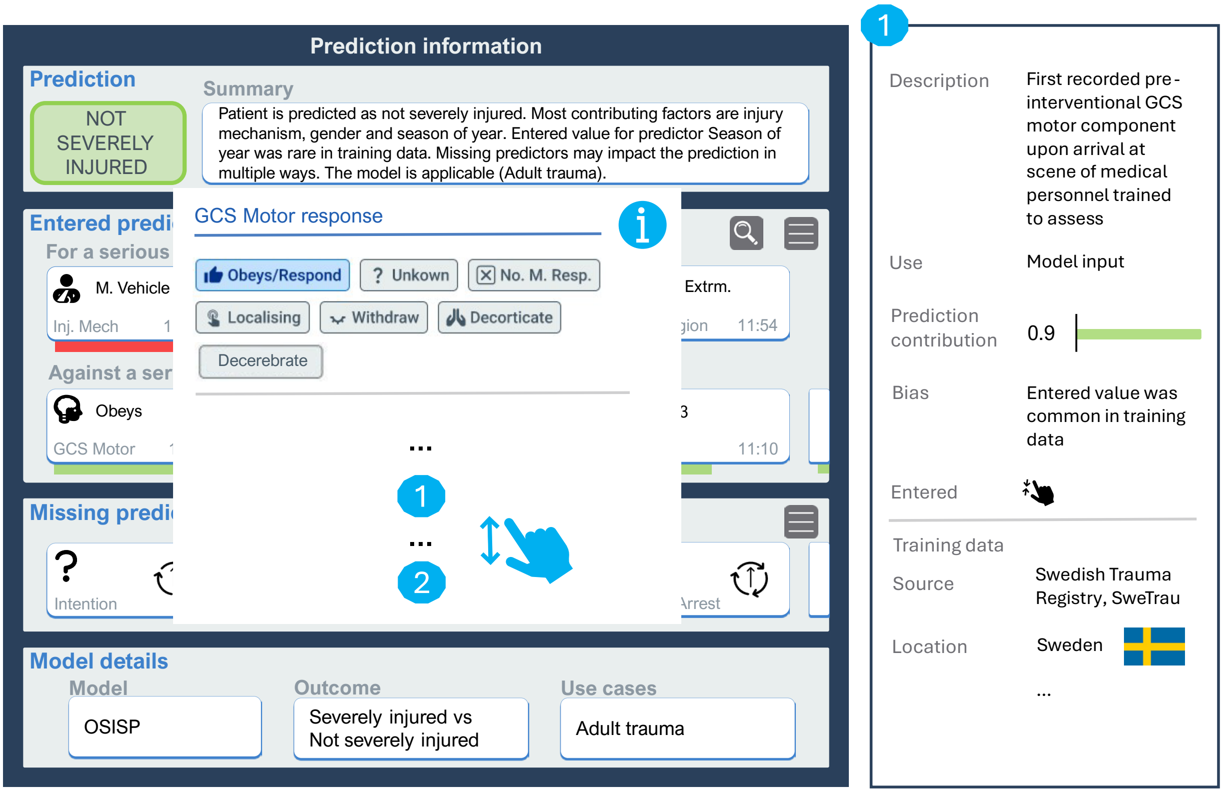
Prediction information
Prediction
NOT SEVERELY INJURED
Summary
Patient is predicted as not severely injured. Most contributing factors are injury mechanism, gender and season of year. Entered value for predictor Season of year was rare in training data. Missing predictors may impact the prediction in multiple ways. The model is applicable (Adult trauma).
GCS Motor response
Obeys/Respond
Unkown
No. M. Resp.
Localising
Withdraw
Decorticate
Decerebrate
1
2
Model details
Model
OSISP
Outcome
Severely injured vs Not severely injured
Use cases
Adult trauma
1
Description
First recorded pre-interventional GCS motor component upon arrival at scene of medical personnel trained to assess
Use
Model input
Prediction contribution
0.9
Bias
Entered value was common in training data
Entered
Training data
Source
Swedish Trauma Registry, SweTrau
Location
Sweden

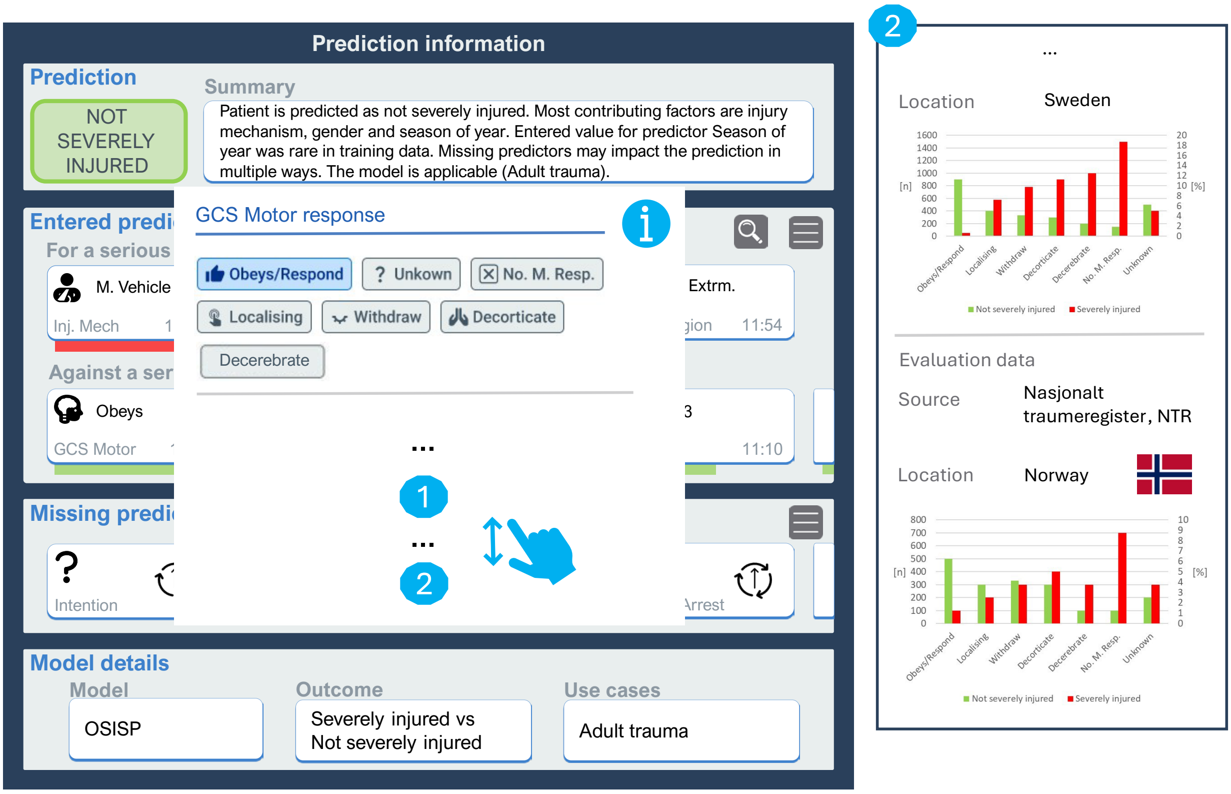
Prediction information
Prediction
NOT SEVERELY INJURED
Summary
Patient is predicted as not severely injured. Most contributing factors are injury mechanism, gender and season of year. Entered value for predictor Season of year was rare in training data. Missing predictors may impact the prediction in multiple ways. The model is applicable (Adult trauma).
Entered predi
For a serious
M. Vehicle
Inj. Mech
Against a ser
Obeys
GCS Motor
Extrm.
11:54
11:10
GCS Motor response
Obeys/Respond
Unkown
No. M. Resp.
Localising
Withdraw
Decorticate
Decerebrate
...
1
2
Missing predi
Intention
Arrest
Model details
Model
OSISP
Outcome
Severely injured vs Not severely injured
Use cases
Adult trauma
2
Location
Sweden
Not severely injured
Severely injured
Evaluation data
Source
Nasjonalt traumeregister, NTR
Location
Norway

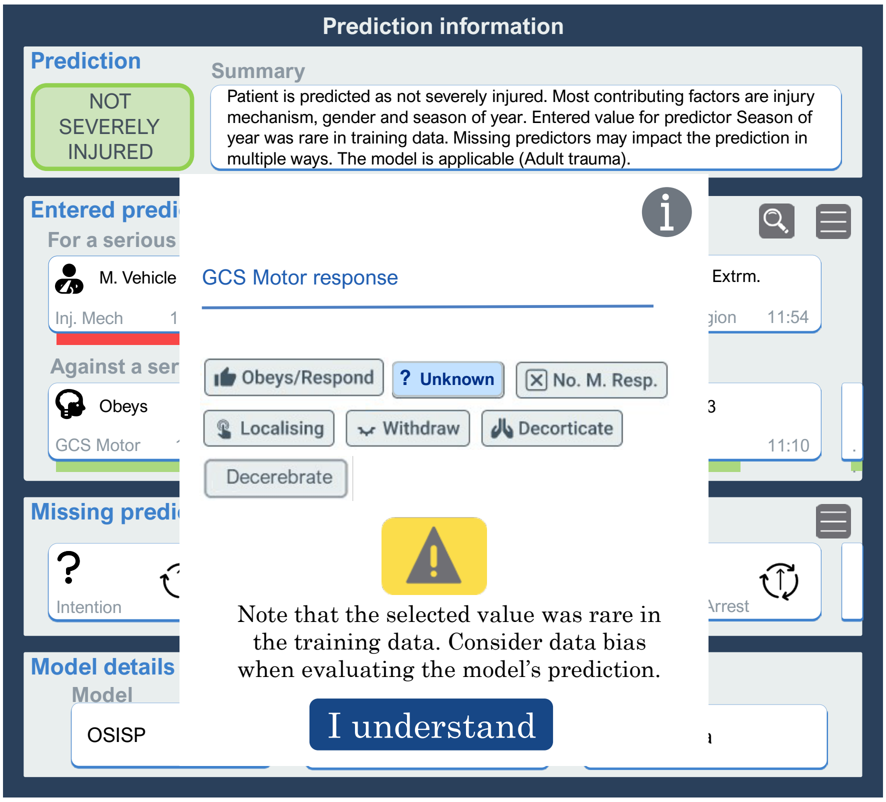
Prediction information
Prediction
NOT SEVERELY INJURED
Summary
Patient is predicted as not severely injured. Most contributing factors are injury mechanism, gender and season of year. Entered value for predictor Season of year was rare in training data. Missing predictors may impact the prediction in multiple ways. The model is applicable (Adult trauma).
Entered predi
For a serious
M. Vehicle
Inj. Mech
Against a ser
Obeys
GCS Motor
Extrm.
11:54
11:10
GCS Motor response
Obeys/Respond
Unknown
No. M. Resp.
Localising
Withdraw
Decorticate
Decerebrate
Note that the selected value was rare in the training data. Consider data bias when evaluating the model's prediction.
I understand
Missing predi
Intention
Arrest
Model details
Model
OSISP

Function 1, Filter of top three predictors (F1)

Function 1 filters entered predictors so that only the top three predictors for and against the condition are displayed.

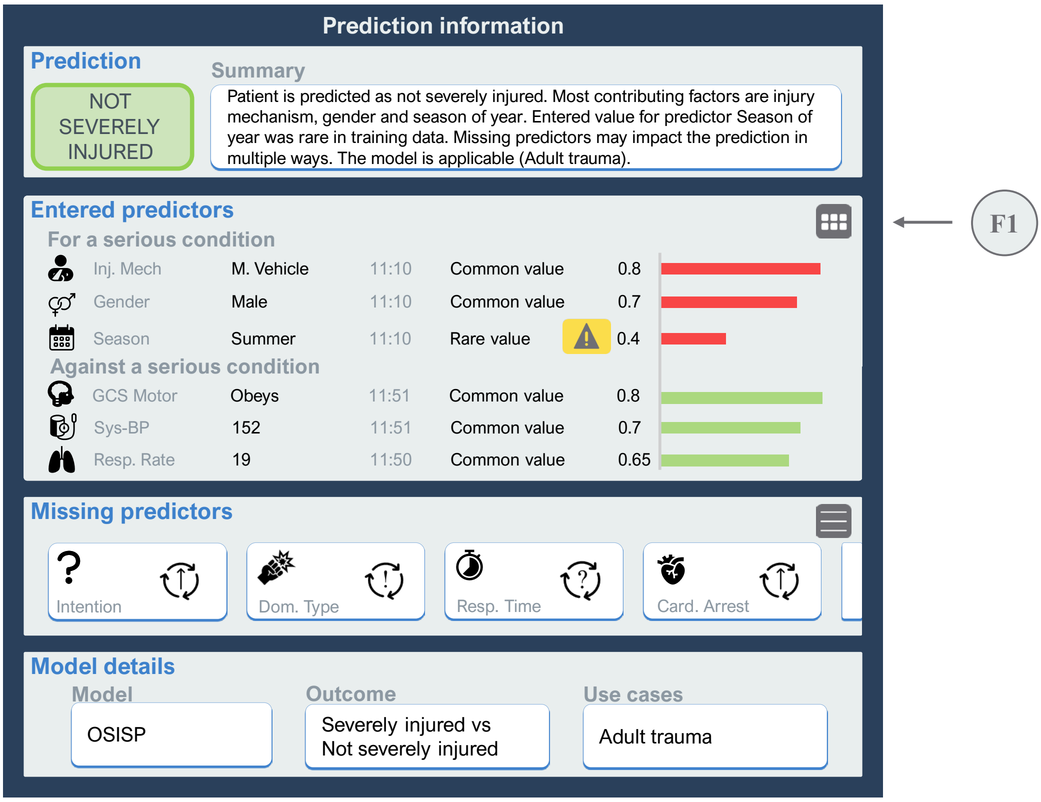

Function 2, Exploration mode with example (F2)

Function 2 enables exploration mode, which freezes the prediction information page. The user may now test different predictor values to explore the impact on the prediction and predictor contributions. An example is displayed below, where the season of year has changed, causing the prediction to change as well as the ranking on predictors for and against the condition.

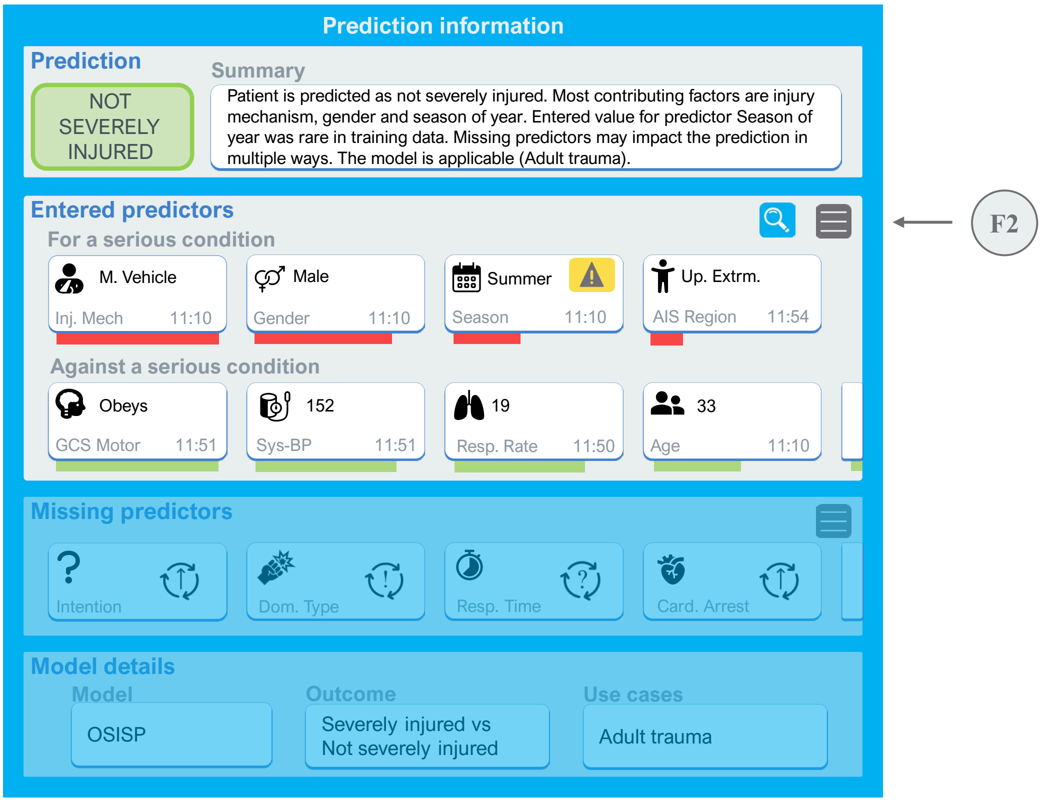

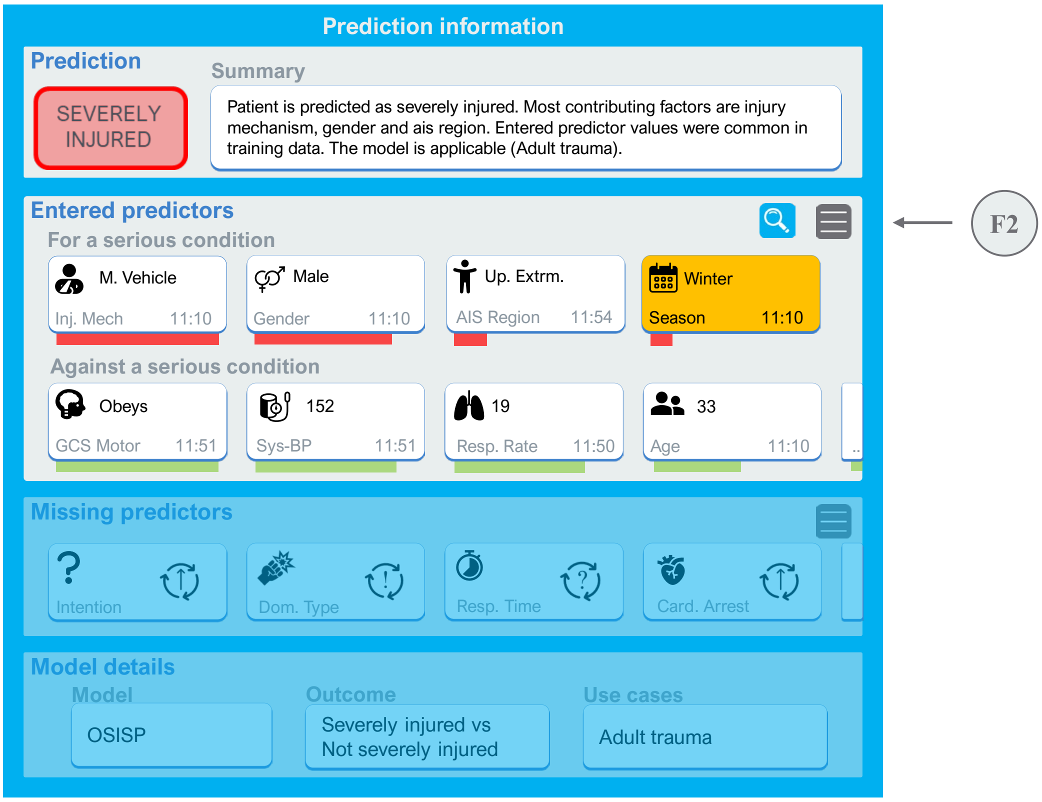

Component 5, Missing predictors (C5): Extended information

Component 5 displays missing/not yet entered predictors, and if any predictor is pressed, a pop-up box with extended information is accessed. The initial pop-up box is similar to component 3 and 4, with addition information on the prediction impact. If the information box is pressed, additional scrollable information is accessed, containing the same information as displayed in the numbered boxes 1–2 for component 3 and 4.

Section 3 C5

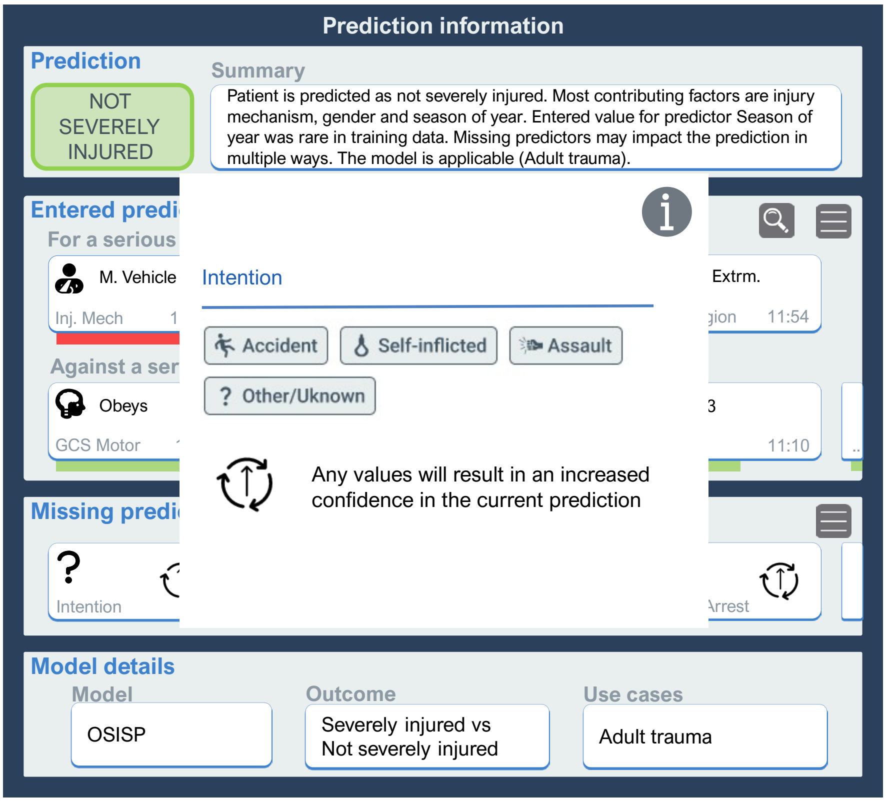

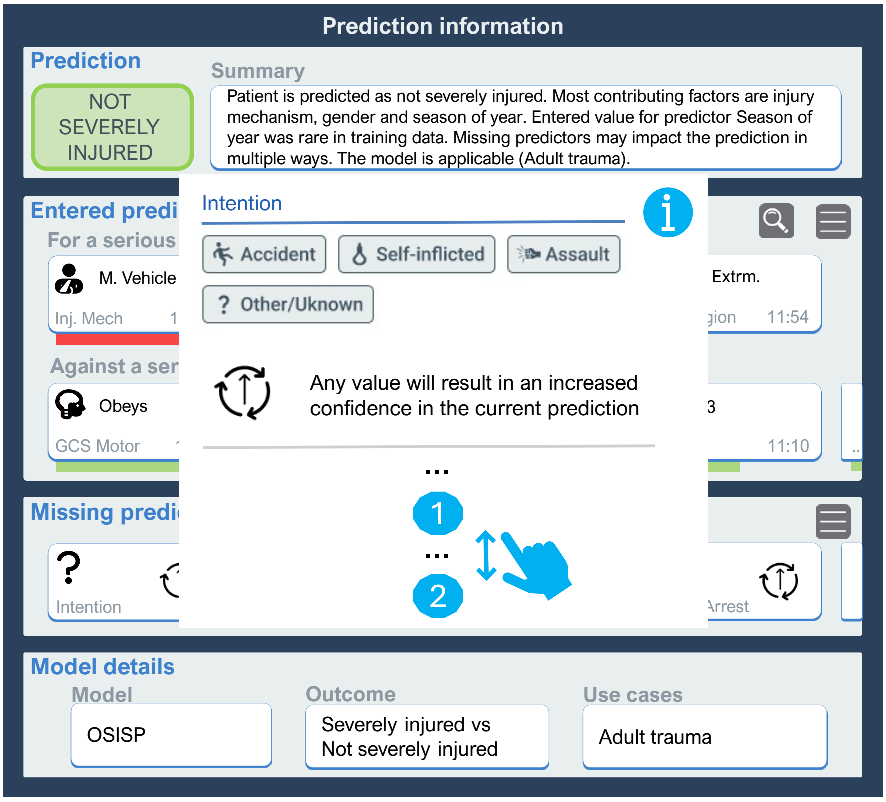

Same extended information as for C3 and C4

Function 3, Filter of top 3 predictors (F3)

Function 3 is similar to function 1 and filters missing predictors so that only the top three predictors with the largest risk of impacting the prediction towards a negative condition, in this case severely injured, are displayed.

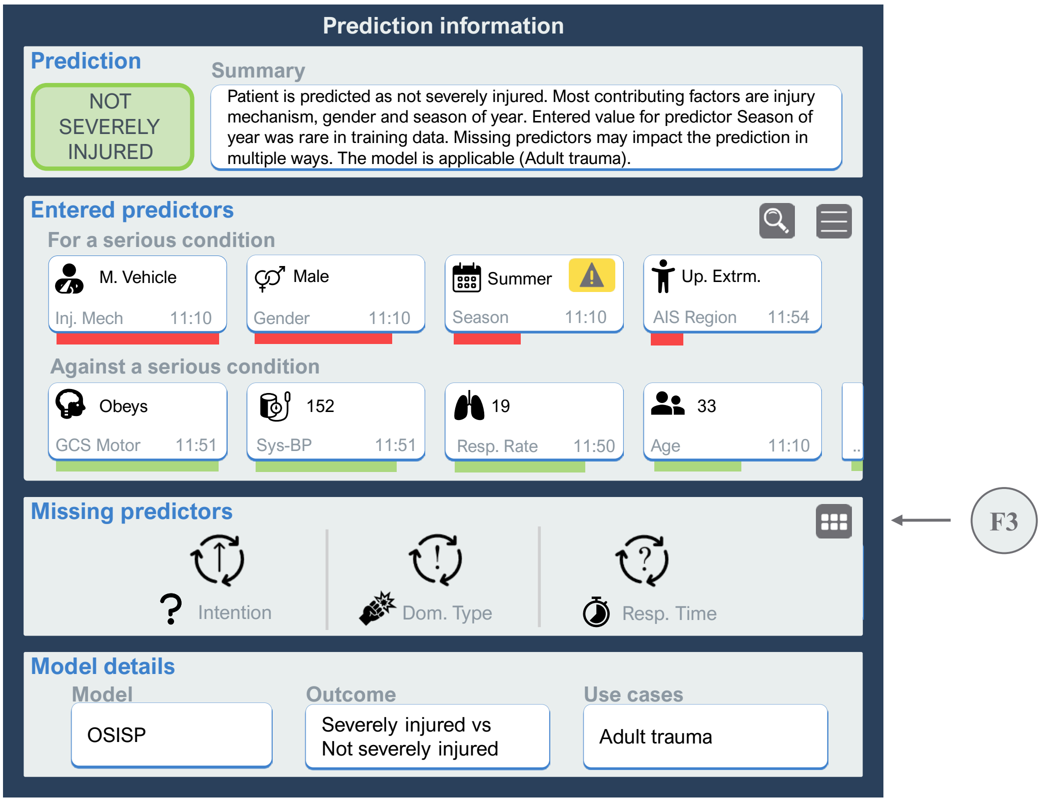

Component 6, Model (C6): Extended information

Component 6 displays the model's name, and if pressed, a pop-up box with extended information is accessed. The pop-up box is scrollable and may access information displayed in the numbered boxes 1–3. If the document symbol is pressed, the user may access complete model and data documentation.

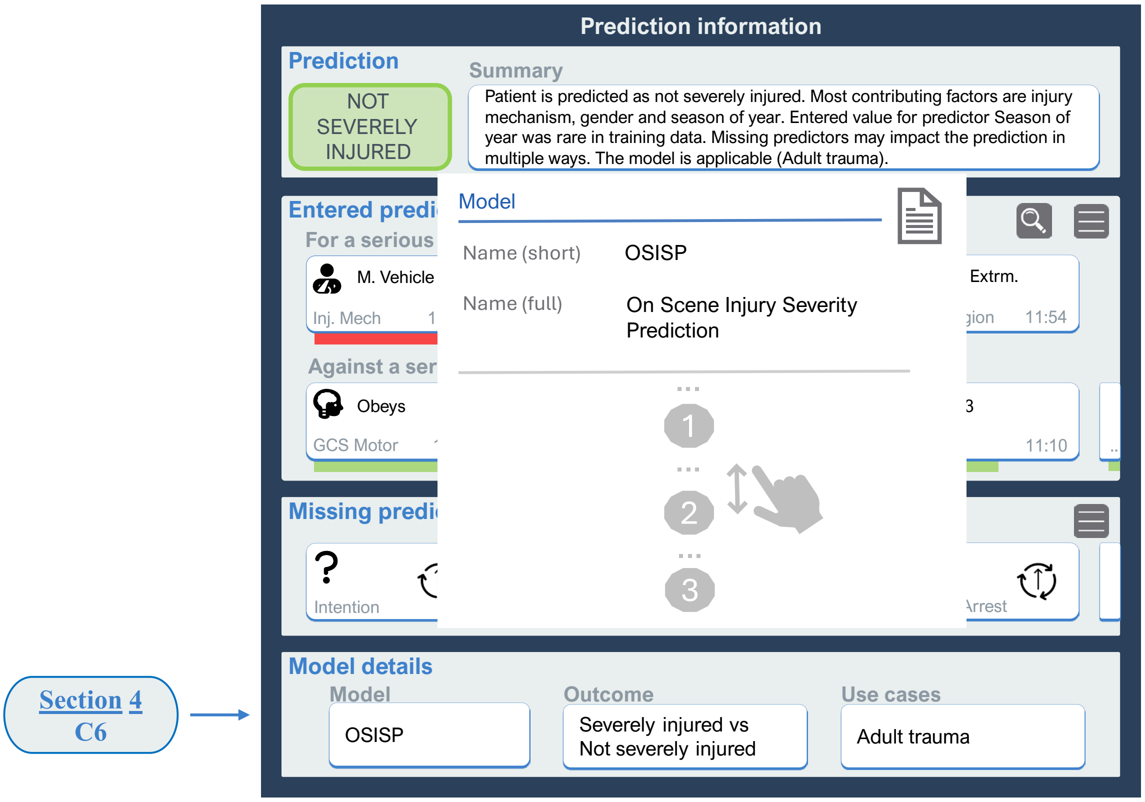
Section 4
C6
Prediction information
Prediction
NOT SEVERELY INJURED
Summary
Patient is predicted as not severely injured. Most contributing factors are injury mechanism, gender and season of year. Entered value for predictor Season of year was rare in training data. Missing predictors may impact the prediction in multiple ways. The model is applicable (Adult trauma).
Entered predi
For a serious
M. Vehicle
Inj. Mech
Against a ser
Obeys
GCS Motor
Extrm.
11:54
11:10
Missing predi
Intention
Arrest
Model
Name (short) OSISP
Name (full) On Scene Injury Severity Prediction
1
2
3
Model details
Model
OSISP
Outcome
Severely injured vs Not severely injured
Use cases
Adult trauma

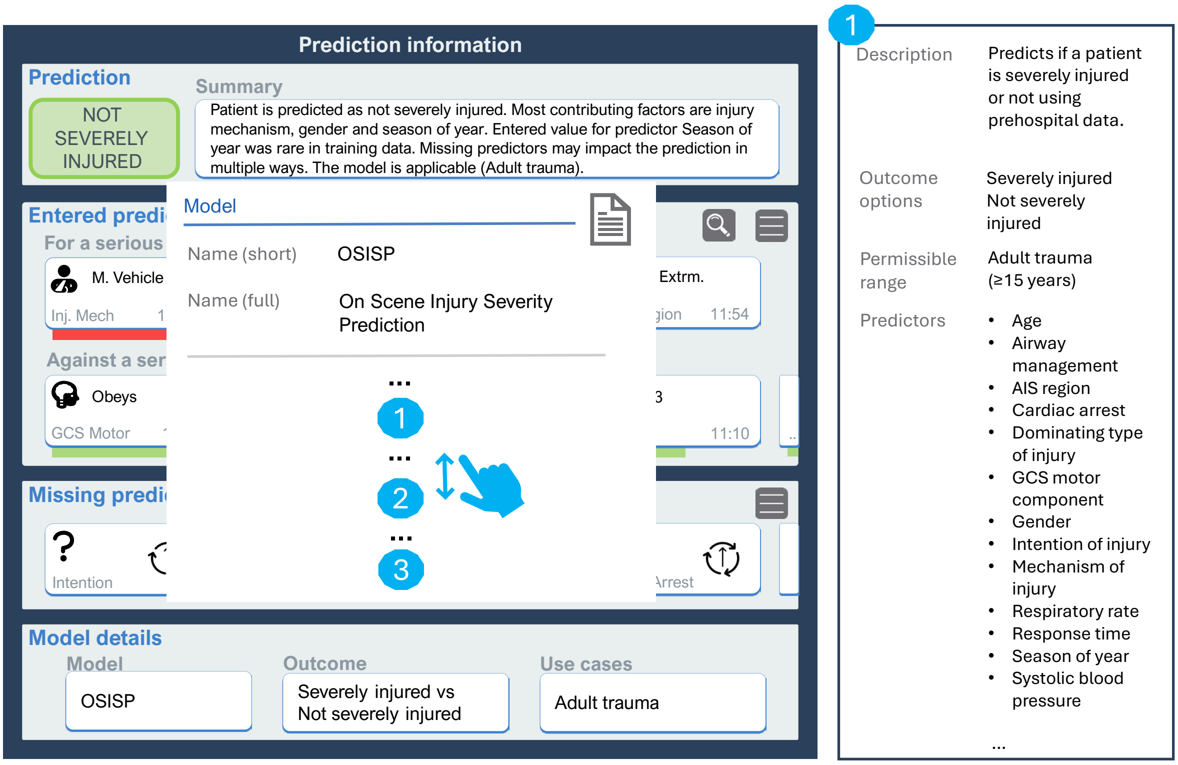
Prediction information
Prediction
NOT SEVERELY INJURED
Summary
Patient is predicted as not severely injured. Most contributing factors are injury mechanism, gender and season of year. Entered value for predictor Season of year was rare in training data. Missing predictors may impact the prediction in multiple ways. The model is applicable (Adult trauma).
Entered predi
For a serious
M. Vehicle
Inj. Mech
Against a ser
Obeys
GCS Motor
Extrm.
11:54
11:10
Missing predi
Intention
Arrest
Model
Name (short) OSISP
Name (full) On Scene Injury Severity Prediction
1
2
3
Model details
Model
OSISP
Outcome
Severely injured vs Not severely injured
Use cases
Adult trauma
1
Description
Predicts if a patient is severely injured or not using prehospital data.
Outcome options
Severely injured
Not severely injured
Permissible range
Adult trauma (≥15 years)
Predictors
Age
Airway management
AIS region
Cardiac arrest
Dominating type of injury
GCS motor component
Gender
Intention of injury
Mechanism of injury
Respiratory rate
Response time
Season of year
Systolic blood pressure
...

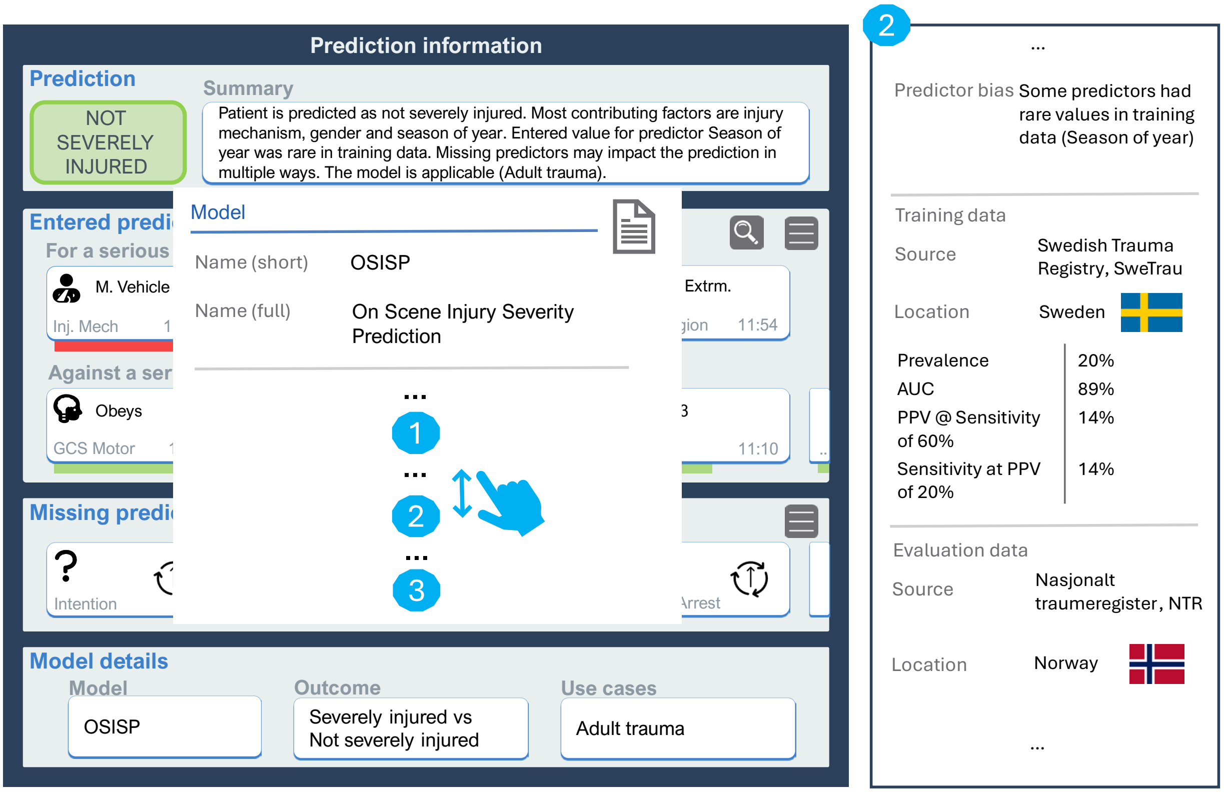

Prediction information
Prediction
NOT SEVERELY INJURED
Summary
Patient is predicted as not severely injured. Most contributing factors are injury mechanism, gender and season of year. Entered value for predictor Season of year was rare in training data. Missing predictors may impact the prediction in multiple ways. The model is applicable (Adult trauma).
Model
Name (short)
OSISP
Name (full)
On Scene Injury Severity Prediction
1
2
3
Model details
Model
OSISP
Outcome
Severely injured vs Not severely injured
Use cases
Adult trauma
2
Predictor bias
Some predictors had rare values in training data (Season of year)
Training data
Source
Swedish Trauma Registry, SweTrau
Location
Sweden
Prevalence 20%
AUC 89%
PPV @ Sensitivity of 60% 14%
Sensitivity at PPV of 20% 14%
Evaluation data
Source
Nasjonalt traumeregister, NTR
Location
Norway

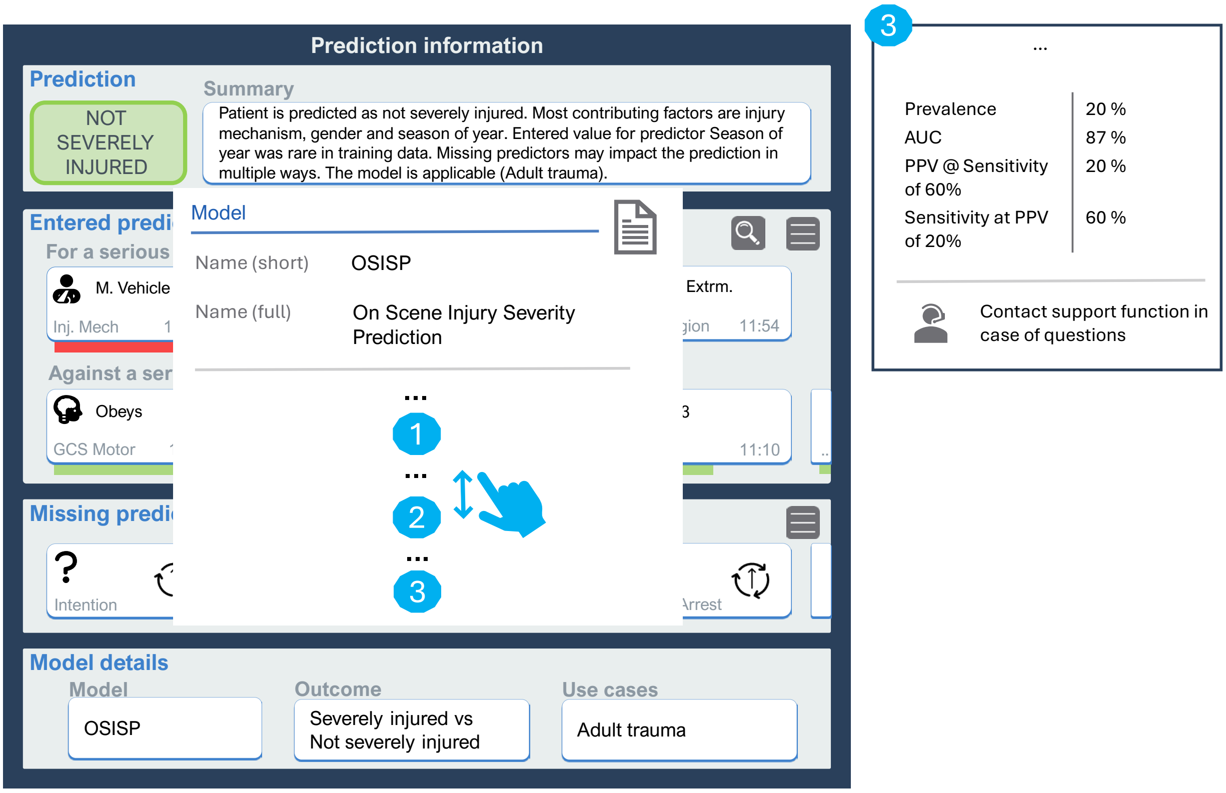

Prediction information
Prediction
NOT SEVERELY INJURED
Summary
Patient is predicted as not severely injured. Most contributing factors are injury mechanism, gender and season of year. Entered value for predictor Season of year was rare in training data. Missing predictors may impact the prediction in multiple ways. The model is applicable (Adult trauma).
Model
Name (short)
OSISP
Name (full)
On Scene Injury Severity Prediction
1
2
3
Model details
Model
OSISP
Outcome
Severely injured vs Not severely injured
Use cases
Adult trauma
3
Prevalence 20 %
AUC 87 %
PPV @ Sensitivity of 60% 20 %
Sensitivity at PPV of 20% 60 %
Contact support function in case of questions

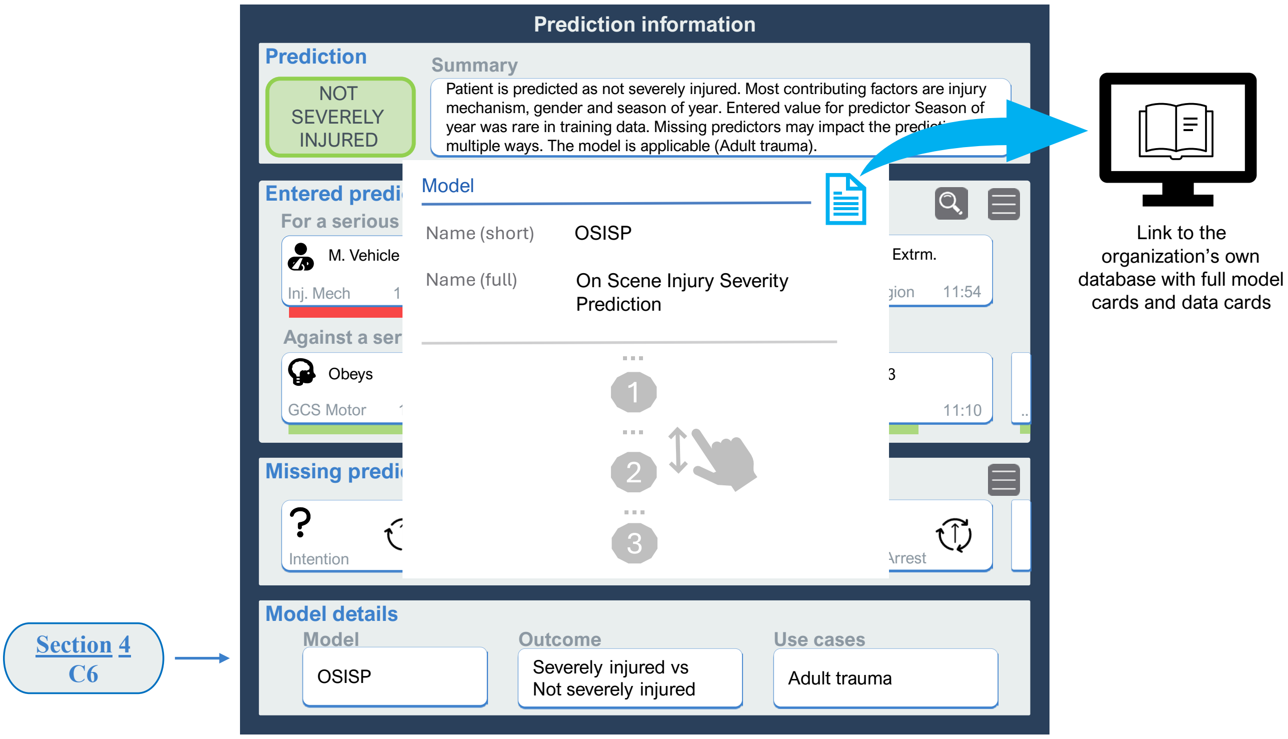

Component 7, Model outcome (C7): Extended information

Component 7 displays the model's outcome range, and if pressed, a pop-up box with extended information is accessed.

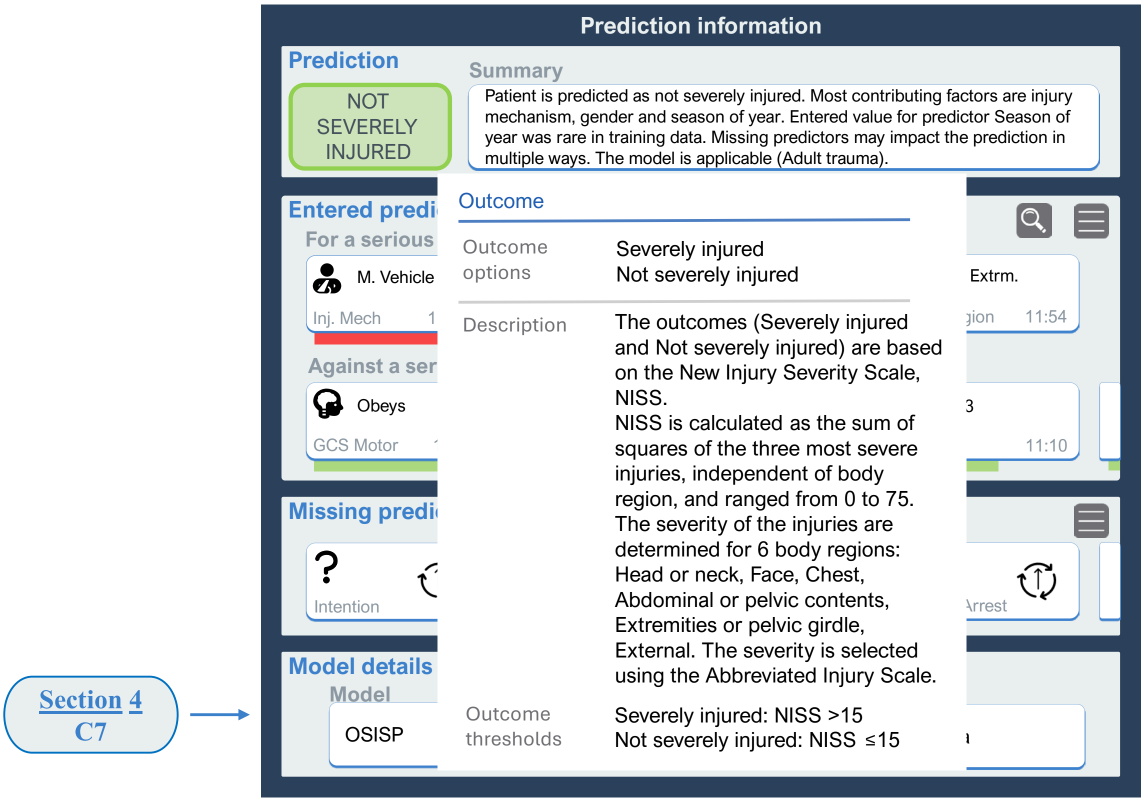

Component 8, Model use cases (C8): Extended information

Component 8 displays when the model is applicable, and if pressed, a pop-up box with extended information is accessed. If an entered predictor value has been entered that violates the use cases, a warning will be communicated to the user and the prediction information page will be blocked until the value has changed.

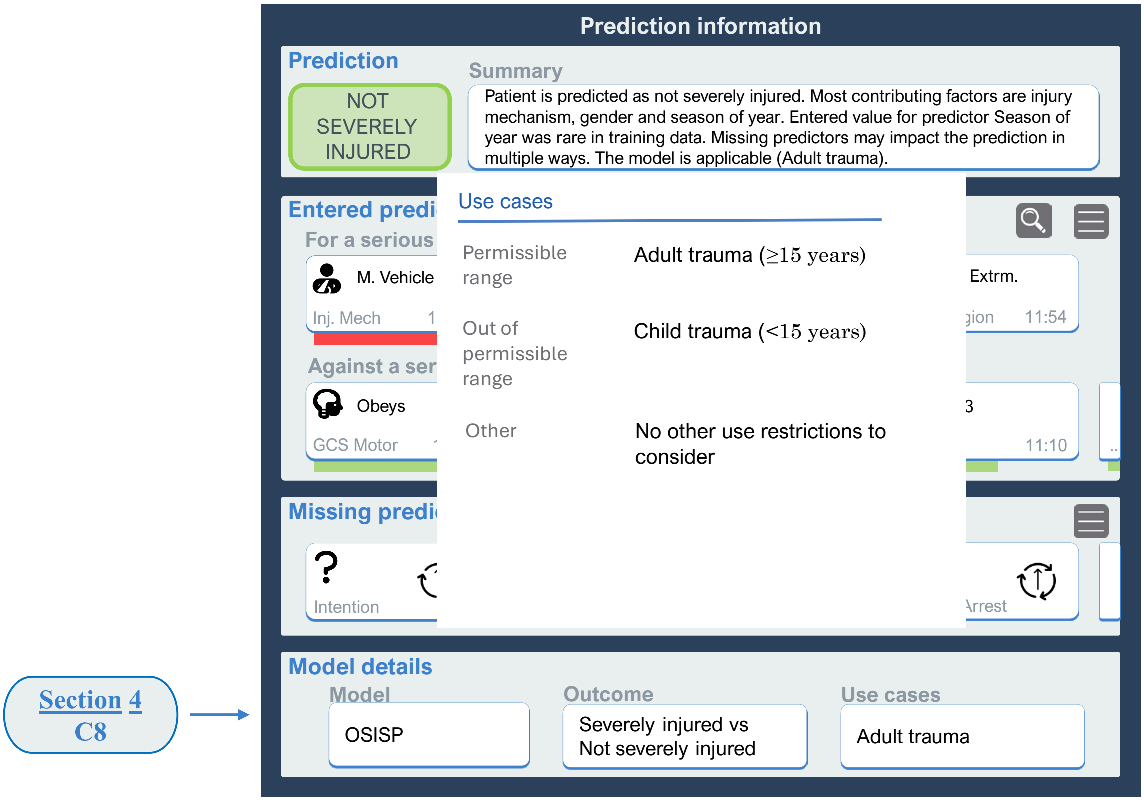

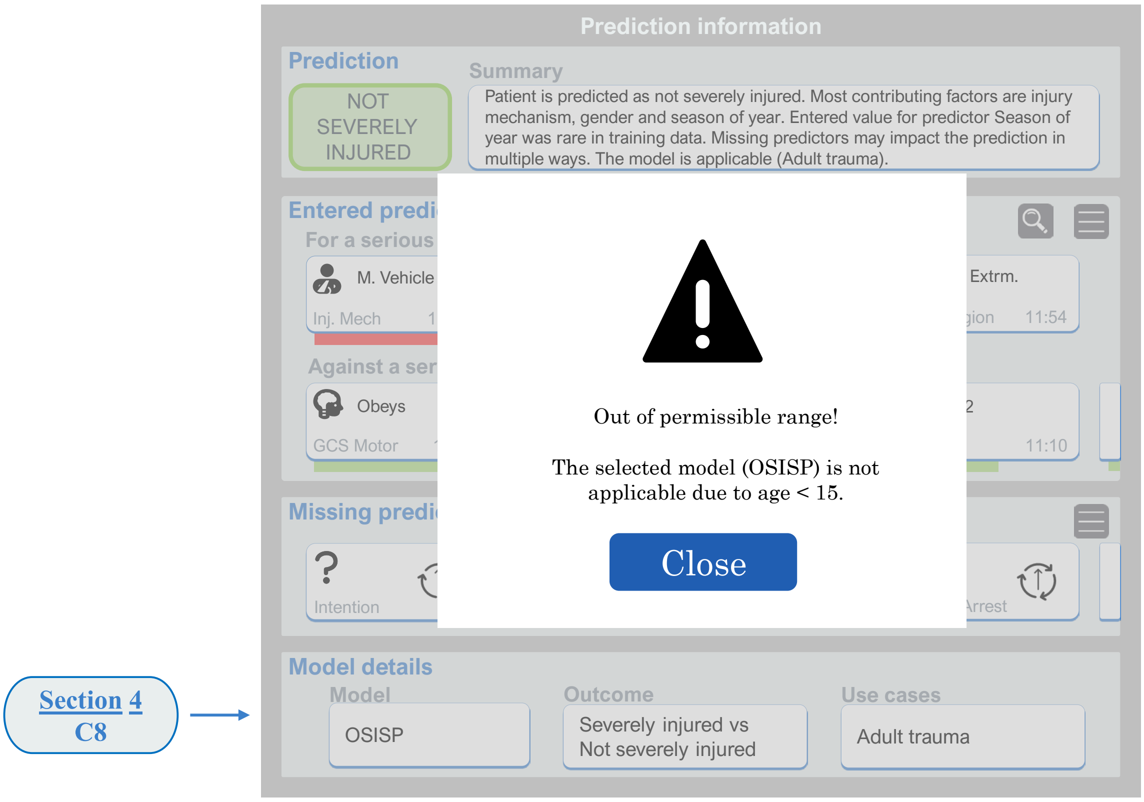

Supplement: sj-pdf-5-dhj-10.1177_20552076251403207 - Supplemental material for Human–AI collaboration for prehospital trauma triage: Designing the On Scene Injury Severity Prediction (OSISP) model as a clinical decision support system [file sj-pdf-5-dhj-10.1177_20552076251403207.pdf]
